# Short-Term and Long-Term Sensitization Differentially Alters the Composition of an Anterograde Transport Complex in *Aplysia*

**DOI:** 10.1523/ENEURO.0266-22.2022

**Published:** 2023-01-03

**Authors:** Abhishek Sadhu, Kerriann K. Badal, Yibo Zhao, Adia A. Ali, Supriya Swarnkar, George Tsaprailis, Gogce C. Crynen, Sathyanarayanan V. Puthanveettil

**Affiliations:** 1 Department of Neuroscience, UF Scripps Biomedical Research, University of Florida, Jupiter, Florida 33458; 2 Integrated Biology Graduate Program, Florida Atlantic University, Jupiter, Florida 33458; 3 Proteomics Core, UF Scripps Biomedical Research, University of Florida, Jupiter, Florida 33458; 4 Bioinformatics Core, UF Scripps Biomedical Research, University of Florida, Jupiter, Florida 33458

**Keywords:** axonal transport, kinesin, learning, long-term sensitization, quantiative proteomics, short-term sensitization

## Abstract

Long-term memory formation requires anterograde transport of proteins from the soma of a neuron to its distal synaptic terminals. This allows new synaptic connections to be grown and existing ones remodeled. However, we do not yet know which proteins are transported to synapses in response to activity and temporal regulation. Here, using quantitative mass spectrometry, we have profiled anterograde protein cargos of a learning-regulated molecular motor protein kinesin [*Aplysia* kinesin heavy chain 1 (ApKHC1)] following short-term sensitization (STS) and long-term sensitization (LTS) in *Aplysia californica*. Our results reveal enrichment of specific proteins associated with ApKHC1 following both STS and LTS, as well as temporal changes within 1 and 3 h of LTS training. A significant number of proteins enriched in the ApKHC1 complex participate in synaptic function, and, while some are ubiquitously enriched across training conditions, a few are enriched in response to specific training. For instance, factors aiding new synapse formation, such as synaptotagmin-1, dynamin-1, and calmodulin, are differentially enriched in anterograde complexes 1 h after LTS but are depleted 3 h after LTS. Proteins including gelsolin-like protein 2 and sec23A/sec24A, which function in actin filament stabilization and vesicle transport, respectively, are enriched in cargos 3 h after LTS. These results establish that the composition of anterograde transport complexes undergo experience-dependent specific changes and illuminate dynamic changes in the communication between soma and synapse during learning.

## Significance Statement

Despite advances in our understanding of mechanisms underlying activity-dependent transport from soma to synapse, the specific gene products transported to synapses during learning are not yet understood in any system. Using quantitative proteomic analysis of an anterogradely transported protein complex, we find that this complex undergoes dynamic compositional changes during short-term and long-term sensitization training. These findings bring new insights into the regulation of soma-to-synapse communication during learning.

## Introduction

Activity-dependent synaptic plasticity is key to the formation of long-term memories (LTMs) and involves changes in the expression of specific genes as well as the synthesis of new proteins. This then results in structural remodeling of the synapse (Martin et al., 2000; Kandel, 2001; Sutton and Schuman, 2006). Mechanistic investigations of LTM in vertebrate and invertebrate animal models have identified different phases of memory formation—an early short-term phase independent of gene expression changes and new protein synthesis, which lasts typically 30 min to 1 h, an intermediate-term phase prolonging for 1–3 h, which requires protein synthesis, but not gene transcription, and a later long-term phase, which lasts >8 h and is sensitive to gene expression disruptions and protein synthesis (Emptage and Carew, 1993; Ghirardi et al., 1995; Sutton and Carew, 2000; Kandel, 2001; Sutton and Schuman, 2006; Byrne and Hawkins, 2015). This alteration in gene expression resulting in synaptic structural changes poses a compelling question in cell biology: how are somatic-to-synaptic processes coordinated and comprised?

We have previously demonstrated in both invertebrate and vertebrate models of LTM that members of the kinesin family of molecular motor proteins (KIFs) are physiologically regulated and play a central role in coordinating communication from soma to synapse (marine sea slug *Aplysia*: Puthanveettil et al., 2008; mouse: Swarnkar et al., 2021 ). KIFs transport gene products as multiprotein RNA complexes along the microtubule cytoskeleton (Kanai et al., 2004; Puthanveettil et al., 2008; Hirokawa et al., 2010; Swarnkar et al., 2021). Several studies including our own have shown that KIF transport carries synaptic vesicle precursors along with synaptic proteins, for example, synaptophysin, synaptotagmin, neuroligin-2, and small GTPase (Okada et al., 1995; Niwa et al., 2008; Liu et al., 2014); fodrin-associated vesicles to assist the growth of neurites (Takeda et al., 2000); NMDA, AMPA, and GABA_A_ receptors (Setou et al., 2002; Twelvetrees et al., 2010; Liu et al., 2014); RNAs and messenger ribonucleoprotein complexes (Kanai et al., 2004; Puthanveettil, 2013; Cross et al., 2021; Fukuda et al., 2021); and organelles such as mitochondria and lysosomes (Nangaku et al., 1994; Nakata and Hirokawa, 1995; Tanaka et al., 1998; Badal et al., 2019).

Such control of synaptic function by KIFs raises an important and unanswered question: do the contents of transported cargo change during learning and LTM? We speculated that identifying the transported proteome during the dynamic process of memory formation would provide insights into the molecules critical for synapse remodeling and/or new synapse formation. Furthermore, how the composition of anterogradely transported proteins is altered during learning could enlighten us to clinically significant mechanisms underlying transport-related neurologic diseases including Alzheimer’s disease, amyotrophic lateral sclerosis, and Parkinson’s disease, as well as Charcot– Marie–Tooth disease (Zhao et al., 2001; Lazarov et al., 2007; Bilsland et al., 2010; Chu et al., 2012; Gunawardena et al., 2013).

To understand the potentially evolving composition of anterograde transport complexes during learning and LTM, we took advantage of the robust learning paradigm of sensitization, a form of nonassociative learning in the sea slug *Aplysia californica*. Specifically, we used sensitization of siphon withdrawal reflex, whereby a single electric tail shock induces short-term sensitization (STS) in the siphon withdrawal response of *Aplysia* lasting 30 min to 1 h. Four repeated tail shocks result in long-term sensitization (LTS), with >24 h of siphon withdrawal (Pinsker et al., 1970, 1973; Carew et al., 1971, 1981; Hawkins et al., 1989; Antonov et al., 1999, 2003). Previously, we reported that the expression of a molecular motor protein kinesin called *Aplysia* kinesin heavy chain 1 (ApKHC1) in sensory and motor neurons of gill withdrawal reflex are upregulated by serotonin, a modulatory neurotransmitter involved in sensitization and learning (Puthanveettil et al., 2008). Given this finding, we here evaluated ApKHC1 cargos following both STS and LTS training in *Aplysia*. We isolated ApKHC1 complexes following training and studied their composition using quantitative mass spectrometry. Using isobaric tandem mass tags (TMTs), we found that 28% of identified anterogradely trafficked proteins participated in synaptic function. A quantitative comparison of cargo protein enrichment among different sensitization conditions demonstrated the presence of activity-dependent regulation of anterograde transport, suggesting that proteins on the transport complex undergo temporal alteration based on the different stages of memory formation.

## Materials and Methods

### Behavioral training and dissection

Eight-month-old *Aplysia* weighing 80–100 g were used for the study. Long-term sensitization training was conducted following the study by Pinsker et al. (1973). Since, sea slugs are hermaphroditic animals, sex-related differentiations are not applicable to this study. Briefly, each animal was housed individually 48 h before behavioral training and maintained in circulating seawater at 18°C. Responsiveness was pretested by light siphon touch using a brush. Animals with a response time of ≥10 s were selected for the experiment. These qualifying animals were then randomly selected into a control (untrained) group, a short-term sensitized (single shock) group, and two long-term sensitized (four shocks) groups, with no significant variance among the groups. Sensitization was induced by delivering four 6 V, 15 mA shocks for 1.5 s every 3 s. The short-term sensitized group received this once, and the long-term sensitized groups received it four times in 30 min intervals. Electrical shocks were delivered on the posterior body wall in the tail region.

**Figure 1.**
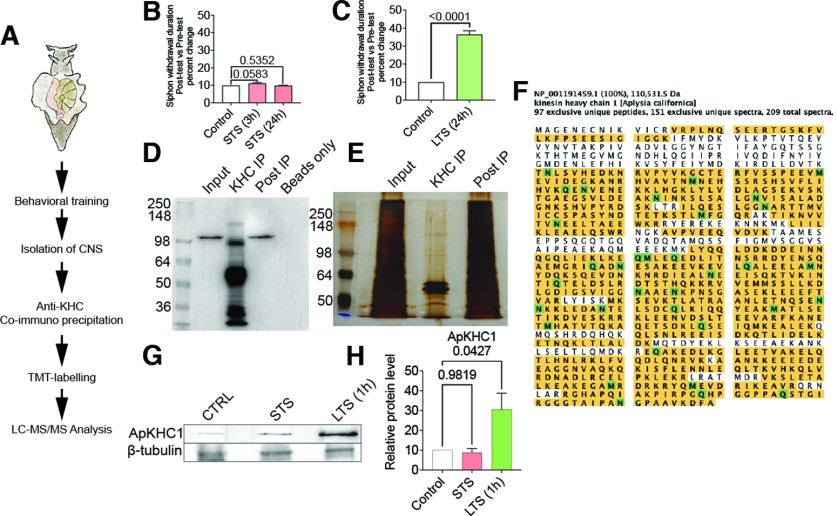
Identification of kinesin complexes in *Aplysia* CNS. ***A***, The approach used to capture ApKHC1-specific protein cargos in *Aplysia* ganglia. The scheme represents the workflow for pulling down the ApKHC1 cargo complex for proteomics. ApKHC1 complexes were immunoprecipitated using an anti-ApKHC1 antibody. ***B***, ***C***, Siphon withdrawal response on STS training (***B***) and LTS training (***C***). Protein complexes were then separated on SDS/PAGE. ***D***, ***E***, IP of ApKHC1 was validated by Western blot analysis (***D***) and silver staining (***E***). ***F***, Image of ApKHC1 amino acid sequence showing ∼74% coverage by 150 tryptic peptides identified in mass spectrometry analysis. ***G***, ApKHC1 levels were studied after 1 h of STS and LTS by Western blot analysis. ***H***, Fold increase in ApKHC1 protein levels after 1 h of STS and LTS. Relative protein levels are expressed as the mean fold change, with error bars showing SEM; statistical analyses were performed by one-way ANOVA followed by Dunnett’s *post hoc* test (Extended Data Fig. 1-1, tables 1–5, Extended Data Fig. 1-2).

The entire CNS (without buccal ganglia) was isolated 1 h after the delivery of single shocks (STS) or four shocks (1 h; LTS) or 3 h after four shocks (3 h; LTS). The percentage change in siphon withdrawal duration post-test versus pretest is provided in Figure 1, *B* and *C* (Extended Data Fig. 1-1, tables 1–5). To anesthetize the animals, an intracoelomic injection of 0.38 m MgCl_2_ was administered. The cerebral, pedal, and pleural ganglia ring was collected with attached abdominal ganglia and, to block synaptic transmission, transferred to ice-cold 1:1 artificial seawater/isotonic MgCl_2_ before lysis.

10.1523/ENEURO.0266-22.2022.f1-1 Figure 1-1 Table 1, Short-term sensitization behavioral training dataset. Table 2, Long-term sensitization behavioral training dataset. Table 3, Antibodies described in this study. Table 4, KHC peptide identified by MS/MS. Table 5, WB analysis of ApKHC1 in response to STS and LTS (1 h). Download Figure 1-1, XLS file.

**Figure 2.**
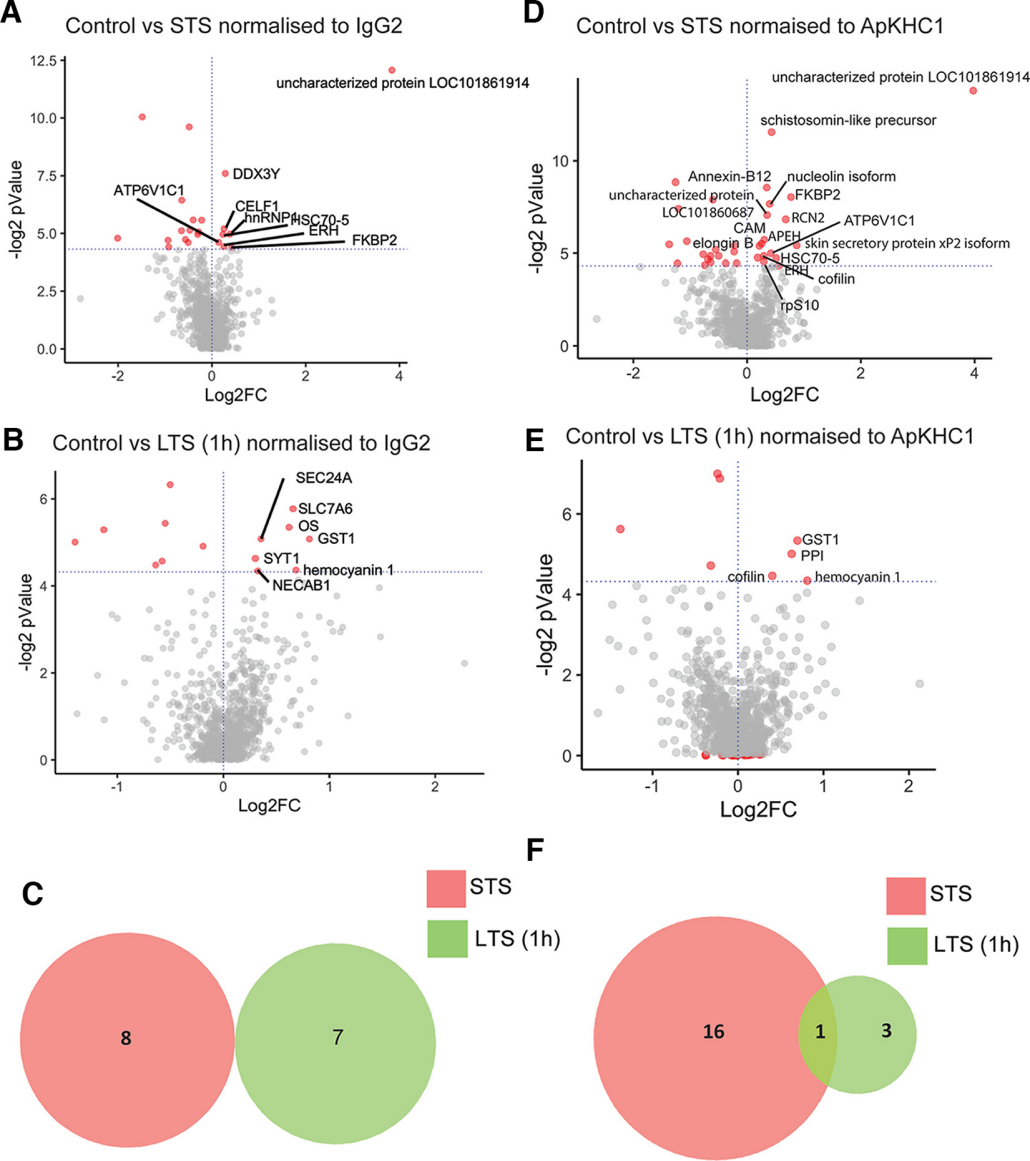
Graphical illustration of quantitative proteomics data showing differential enrichment of protein sets after STS and LTS (1 h). Proteins are ranked in a volcano plot according to their statistical -log2 *p*-value (*y*-axis) and their relative abundance ratio (log2 fold change) between higher enrichment and depletion (*x*-axis). Red dots indicate significantly regulated proteins (false discovery rate, <0.01; s0 = 1). ***A***, Volcano plot of control versus STS protein enrichment normalized to IgG2. ***B***, Volcano plot of control versus LTS (1 h) protein enrichment normalized to IgG2. ***C***, Venn diagram comparison of sets of proteins showed higher enrichment in response to STS and LTS (1 h) normalized to IgG2 (*p *<* *0.05). ***D***, Volcano plot of control versus STS protein enrichment normalized to ApKHC1. ***E***, Volcano plot of control versus LTS (1 h) protein enrichment normalized to ApKHC1. ***F***, Venn diagram showing a comparison of sets of proteins showed higher enrichment in response to STS and LTS (1 h) normalized to KHC1 (*p *<* *0.05; Extended Data Fig. 2-1, tables 1–6, Extended Data Fig. 2-2).

10.1523/ENEURO.0266-22.2022.f1-2 Figure 1-2 ***A***, ***B***, Optimization of ApKHC1 IP with different anti-ApKHC1 antibody concentrations. ***C***, Analysis of proteins that are selectively associated with ApKHC1 kinesin complexes. Protein functions were assigned to a group manually checking their biological role. ***D***, GO network plot of identified kinesin cargo based on protein domains (Pfam). ***E***, Predicted structure of helix-loop-helix EF hand motif uncharacterized protein LOC101861914. ***F***, The structure is based on Template 3e3r.1.B Calcyphosin and showed 21.43% sequence identity with LOC101861914 (www.swissmodel.expasy.org). Download Figure 1-2, TIF file.

### ApKHC1 complex isolation and Western blot analysis

For each *N* = 1, four CNSs (*n* = 4) were pooled together and lysed to extract one final sample in a buffer containing 50 mm Tris, pH 7.5, 1 mm EDTA, 150 mm NaCl, 1 mm DTT, 0.5% NP-40, EDTA-free protease inhibitor tablet (Roche), and phosphatase inhibitor cocktails 2 and 3 (Sigma-Aldrich) at 4°C in individual Dounce homogenizers. Lysates were spun down at 10,600 × *g* for 10 min at 4°C. Supernatants were incubated in protein A/G agarose beads, pre-equilibrated, and blocked with lysis buffer for 2 h to control any nonspecific binding of unwanted proteins. After collecting the supernatant by brief centrifugation at 100 × *g*, anti-ApKHC1 antibody (Extended Data Fig. 1-1, tables 1–5) was added and incubated/rotated for 12–14 h. Protein A/G beads were added to the supernatants and incubated for 1 h. The bead–antibody complex was collected and washed three times by brief centrifugation. For SDS-PAGE analysis, complexes were eluted in 2× Laemmli buffer, and for mass spectrometric analysis, they were eluted in 5% SDS (v/v). To carry out Western blot (WB) analyses, the protein concentration was determined using a Qubit protein assay kit (Thermo Fisher Scientific). Approximately 100 μg of protein was loaded onto SDS-PAGE gels and used for WB analyses. The antibodies used are listed in Extended Data Figure 1-1 (tables 1–5). The target protein bands were detected using HRP-conjugated anti-rabbit or anti-mouse secondary antibodies (Cell Signaling Technology) at a 1:5000 dilution followed by visualization using chemiluminescence (GE Health Care). The signal bands were analyzed by ImageJ.

### TMT quantitative proteomics and mass spectrometry

Samples in 5% SDS (v/v) were brought to 24 μl with an additional 5% SDS and processed for digestion using micro-S-Traps (Protifi) according to manufacturer instructions. Briefly, proteins in 5% SDS were reduced with 1 μl of 120 mm TCEP [Tris (2-carboxyethyl) phosphine hydrochloride] at 56°C for 20 min, followed by alkylation using 1 μl of 500 mm methyl methanethiosulfonate for 20 min at ambient temperature. Finally, 4 μg of sequencing-grade trypsin was added to the mixture and incubated for 1 h at 47°C. Following this incubation, 40 μl of 50 mm TEAB (tetraethylammonium bromide) was added to the S-Trap, and the peptides were eluted using centrifugation. Elution was repeated once. A third elution using 35 μl of 50% acetonitrile was also performed, and the eluted peptides were dried under a vacuum. The peptides were subsequently resolubilized in 30 μl of 50 mm triethylammonium bicarbonate, pH 8.5, labeled with TMT labels (10-plex) according to the manufacturer instructions (Thermo Fisher Scientific), and pooled. The multiplexing strategy is provided in Extended Data Figure 2-1 (tables 1–6). The pooled, plexed samples were then dried under vacuum, resolubilized in 1% trifluoroacetic acid, and finally desalted using 2 μg capacity ZipTips (Millipore) according to manufacturer instructions. Peptides were then online eluted into a Fusion Tribrid Mass Spectrometer (Thermo Fisher Scientific) from an EASY PepMap RSLC C18 column (2 μm, 100 Å, 75 μm × 50 cm; Thermo Fisher Scientific), using a gradient of 5–25% solvent B (80:20 acetonitrile/water; 0.1% formic acid) for 180 min, followed by 25–44% solvent B in 60 min, 44–80% solvent B in 0.1 min, a 5 min hold of 80% solvent B, a return to 5% solvent B in 0.1 min, and finally a 10 min hold of solvent B. All flow rates were 250 nl/min delivered using a nEasy-LC1000 nano-liquid chromatography system (Thermo Fisher Scientific). Solvent A consisted of water and 0.1% formic acid. Ions were created at 1.7 kV using an EASY Spray source (Thermo Fisher Scientific) held at 50°C.

10.1523/ENEURO.0266-22.2022.f2-1 Figure 2-1 Table 1, Kinesin heavy chain Ab TMT mass spectrometry plexing strategy. Table 2, Total number of proteins identified by TMT mass spectrometry. Table 3, Raw IgG2-normalized data: comparison of detected peptides Control versus STS. Table 4, Raw IgG2-normalized data: comparison of detected peptides Control versus LTS (1 h). Table 5, Raw kinesin heavy chain 1-normalized data: comparison of detected peptides Control versus STS. Table 6, Raw kinesin heavy chain 1-normalized data: comparison of detected peptides Control versus LTS (1 h). Download Figure 2-1, XLS file.

A synchronous precursor selection (SPS)-MS3 mass spectrometry method was selected based on the work of Ting et al. (2011). Scans were conducted between 380 and 2000 mass/charge ratio (m/z) at a resolution of 120,000 for MS1 in the Orbitrap mass analyzer at an AGC target of 4E5 and a maximum injection of 50 ms. We then performed collision induced dissociation (CID) in the linear ion trap of peptide monoisotopic ions with a charge 2–8 above an intensity threshold of 5E3, using quadrupole isolation of 0.7 m/z and a CID energy of 35%. The ion trap AGC target was set to 1.0E^4^ with a maximum injection time of 50 ms. Dynamic exclusion duration was set at 60 s, and ions were excluded after one time within the ±10 ppm mass tolerance window. The top 10 MS2 ions in the ion trap between 400 and 1200 m/z were then chosen for high-energy collisional dissociation at 65% energy. Detection occurred in the Orbitrap at a resolution of 60,000, an AGC target of 1E5, and an injection time of 120 ms (MS3). All scan events occurred within a 3 s specified cycle time.

### Proteomic data processing and statistical analysis

Quantitative analysis of the TMT experiments was performed simultaneously with protein identification (ID) using Proteome Discoverer 2.5 software. The precursor and fragment ion mass tolerances were set to 10 ppm and 0.6 Da, respectively. Trypsin enzyme was used with a maximum of two missed cleavages. The following databases were used: NCBI identical protein group for *A. californica* (txid6500), a common contaminant FASTA, and P01867 (IgG2b) FASTA files. A SEQUEST search was performed, and the Percolator feature of Proteome Discoverer 2.5 was used to set a false discovery rate of 0.01. The impurity correction factors obtained from Thermo Fisher Scientific for each kit were included in the search and quantification. The following settings were used to search the phosphor-enriched data: dynamic modifications of oxidation/+15.995 Da (M), and deamidated/+0.984 Da (N, Q); and static modifications of TMT6plex/+229.163 Da (N terminus, K) and carbamidomethyl/+57.021 Da (C). Only unique + Razor peptides were considered for quantification purposes. Coisolation threshold and SPS Mass Matches threshold were set to 50 and 65, respectively. Normalization was achieved using P01867 values in Proteome Discoverer 2.5. The normalized abundance values for master proteins were imported into JMP PRO 15.2.0 (SAS), and the resulting 824 proteins were analyzed by *t* test for unequal variances per comparison (control vs STS/LTS 1 h/LTS 3 h). To make sure that variations in the coimmunoprecipitations (co-IPs) are not because of different amounts of antibodies we used, we normalized to IgG levels in the co-IPs. We also considered the possibility that different amounts of cargos might be loaded to the same kinesin complex under different conditions. If this is true, we should observe different amounts of proteins (more or less of the same protein, and or different proteins) in the complex. We therefore also normalized to kinesin levels in the IPs.

### Quantitative real-time PCR analysis

Quantitative real-time PCR (qRT-PCR) analyses were conducted following our previously stated protocols (Liu et al., 2014; Badal et al., 2019). RNA isolation was conducted using TRIzol after 1 h of STS and LTS training from the entire CNS of the trained animals (*n* = 2). Quantification of each transcript is normalized to the *Aplysia* 18S rRNA reference gene following the 2^−ΔΔCt^ method (Livak and Schmittgen, 2001).

### Statistical analyses

Statistical analyses were performed in R and Prism 9 (details provided in the Extended data Figs. 1-1, 2-1, 3-1, 4-1, 5-1). Statistical tests performed were unpaired two-tailed Student’s *t* test and one-way ANOVA followed by Dunnett’s *post hoc* test unless indicated otherwise. The results are graphically represented as the mean ± Standard error of the mean (SEM) throughout the text, unless otherwise stated. N represents the number of independent samples for each experiment.

## Results

### Isolation of kinesin transport complexes using anti-kinesin heavy chain antibody and TMT labeling

To determine whether compositions of anterograde transport complexes are altered during learning and used in activity-associated remodeling of synapses, we established co-IP of ApKHC1 complexes from *Aplysia* CNS (Extended Data Fig. 1-2). Following the study by Puthanveettil et al. (2008) and Liu et al. (2014) and using an anti-KHC1 monoclonal antibody, we established the ApKHC1 co-IP and assessed the efficiency of the IP by silver-staining analysis followed by mass spectrometry and WB (Fig. 1*A*,*D*,*E*). Immunoprecipitated beads alone served as the specificity control in the co-IP. Specific protein bands, corresponding to ApKHC1 and present only in the IP, were not detected in the “beads only” control in the WB. We therefore isolated the corresponding protein bands from the polyacrylamide gel for ApKHC1 and analyzed them by mass spectrometry to identify the band in detail. A NCBI database search of the liquid chromatography tandem mass spectrometry (LC-MS/MS) data against the IPG (Identical Protein Group) database for *A. californica* (txid6500) was conducted. More than 150 tryptic peptides with ∼74% coverage were identified as corresponding to ApKHC1, suggesting the robustness of the IP (Fig. 1F, Extended Data Fig. 1-1, tables 1–5).

As mentioned above, and following previously described methods (Pinsker et al., 1973; Frost et al., 1985; Hawkins et al., 1989; Antonov et al., 1999, 2003), we induced STS in sea slugs by single tail shock, and LTS by four spaced tail shocks. Single-shock training only gives rise to short-term memory and does not induce transcriptional changes in the same way that four tail shocks do (Kandel, 2001), so we decided to compare how anterograde transport is influenced by short-term versus long-term sensitization.

We first examined whether ApKHC1 expression levels are modulated by LTS training. Similar to previously reported changes in ApKHC1 levels induced by 5x5HT in culture (Puthanveettil et al., 2008), we found that LTS training induced a significant increase in ApKHC1 protein levels (fold increase: approximately twofold; *n* = 3; *p *<* *0.05, one-way ANOVA followed by Dunnett’s *post hoc* test; Fig. 1*G*,*H*, Extended Data Fig. 1-1, tables 1–5). Importantly, STS training did not alter ApKHC1 levels.

We next established quantitative proteomic analyses of the ApKHC1 complexes. After 1 h of behavioral training, we prepared five biological replicates of ApKHC1 co-IPs from control and sensitized groups, each containing CNSs from four animals. After tryptic digestion, peptides were labeled with TMTs, multiplexed, and then subjected to LC-MS/MS (detailed methodology found in the Materials and Methods; Extended Data Fig. 2-1, tables 1–6). In total, Proteome Discoverer 2.5 identified >1000 peptides present in the ApKHC1 IPs as possible cargos using the IPG database, which contained 23,501 entries (August 11, 2021; txid6500), with the list of the identified proteins provided in Extended Data Figure 2-1 (tables 1–6). In this selection, ∼28% of proteins have synaptic localization and function (Extended Data Fig. 1-2). Glutamate receptor 3 (XP_012945575.1), ras-related proteins (e.g., XP_005095256.1, XP_005110286.1, XP_012941242.1, XP_005113070.1), synaptobrevin (NP_001191557.1), synaptotagmin-1 (P41823.2), catalytic subunit of protein kinase A (CAA45015.1), and calmodulin (XP_005095389.1) are all examples of synaptic proteins enriched in the ApKHC1 complex. Furthermore, 7B2 precursor (NP_001191628.1), a disintegrin and metalloproteinase with thrombospondin motifs 3 (XP_005110846.3), acidic leucine-rich nuclear phosphoprotein 32 family member B isoform (XP_005112116.1), allograft inflammatory factor 1 (XP_005111045.2), and α-2-macroglobulin (XP_005104186.2) are a few of the proteins enriched in the anterograde complex that are cardinal in neurologic diseases. RNA-binding protein 1 (XP_005108171.1), lark (XP_005103761.1), Musashi homolog 2 (XP_005105059.2), nova-1 isoform (XP_005106815.1), and major vault protein (XP_005108623.1) are examples of RNA-binding proteins. Finally, 5′−3′ exoribonuclease 2 (XP_012934776.1), 6-phosphogluconate dehydrogenase (XP_005109585.1), decarboxylating (XP_005109585.1), and barrier-to-autointegration factor (XP_005106507.1) are some of the nuclear proteins enriched in the ApKHC1 complex.

### Short-term and long-term sensitization training results in the enrichment of distinct proteins in the ApKHC1 complex

For the comparative quantitative analysis of the enriched proteins following STS and LTS training, we normalized the data with Ig γ-2B chain C region (P01867) using an equal amount of anti-ApKHC1 antibody for all IP experiments. Data presented in volcano plots in Figure 2, *A* and *B*, indicate the enriched proteins compared with control on STS and LTS, respectively. Following the IPG database of txid6500, we learned that 3.5% of the total *A. californica* proteome is associated with the ApKHC1 complex.

10.1523/ENEURO.0266-22.2022.f2-2 Figure 2-2 Venn diagrams comparing the total number of proteins differentially enriched in ApKHC1 cargos in samples of STS and LTS (1 h). ***A***, Dataset normalized to IgG2. ***B***, Dataset normalized to ApKHC1. Download Figure 2-2, TIF file.

Differential analysis identified FK506-binding protein 2 (FKBP2; XP_005099972.1), heterogeneous nuclear ribonucleoprotein 1 (hnRNP1; XP_005095623.1), ATP-dependent RNA helicase (DDX3Y; XP_005110572.1), CUGBP Elav-like family member 1 isoform (CELF1; XP_035825722.1), enhancer of rudimentary homolog (ERH; XP_005097025.1), heat shock 70 kDa protein cognate 5 (HSC70-5; XP_005098119.1), V-type proton ATPase subunit C 1-A (ATP6V1C1; XP_005098193.1), and an uncharacterized protein LOC101861914 (XP_005109882.2) as being significantly enriched on STS (Table 1, Extended Data Fig. 2-1, tables 1–6, volcano plots). On the other hand, glutathione *S*-transferase 1 (GST1; XP_012944480.1), hemocyanin 1 (DAC71534.1), probable RNA polymerase II nuclear localization protein (SLC7A6; XP_005102062.1), angiopoietin-related protein 7-like (ANGPTL7; XP_012938614.1), protein transport protein Sec24A (Sec24A; XP_012934788.1), N-terminal EF-hand calcium-binding protein 1 isoform (NECAB1; XP_035827419.1), and synaptotagmin-1 (SYT1; P41823.2) were significantly enriched after 1 h of LTS (Table 1, Extended Data Fig. 2-1, tables 1–6). These data suggest that different sets of proteins were enriched in response to STS versus LTS (Fig. 2*C*). However, analysis of global changes (Extended Data Fig. 2-2) showed that behavioral training also led to depletion of specific protein cargos on STS and LTS as well as 60S ribosomal protein L8, 60S ribosomal protein L44, and ubiquitin, commonly on both STS and LTS (Table 2).

**Table 1:** Protein cargos enriched in ApKHC1 complex following STS and LTS_1 h compared with control normalized to IgG (*p* < 0.05)

Protein cargos enriched on STS		Protein cargos enriched on LTS_1 h	
Description	Abundance ratio: (STS)/(Control)	Description	Abundance Ratio: (LTS_1 h)/(Control)
Uncharacterized protein LOC101861914	14.32	Glutathione *S*-transferase 1	1.755
FK506-binding protein 2	1.341	Hemocyanin 1	1.606
Heterogeneous nuclear ribonucleoprotein 1	1.304	Probable RNA polymerase II nuclear localization protein SLC7A6OS	1.578
ATP-dependent RNA helicase DDX3Y isoform X1	1.218	Angiopoietin-related protein 7-like	1.538
CUGBP Elav-like family member 1 isoform X1	1.196	Protein transport protein Sec24A	1.278
Enhancer of rudimentary homolog	1.19	N-terminal EF-hand calcium-binding protein 1 isoform X1	1.252
Heat shock 70 kDa protein cognate 5	1.176	Synaptotagmin-1	1.233
V-type proton ATPase subunit C 1-A	1.102		

**Table 2:** Protein cargos depleted in ApKHC1 complex following STS and LTS_1 h compared with control normalized to IgG (*p* < 0.05)

Protein cargos depleted on STS		Protein cargos depleted on LTS_1 h	
Description	Abundance ratio: (STS)/(Control)	Description	Abundance Ratio: (LTS_1 h) /(Control)
β-Arrestin-1	0.859	Fibrillin-3 isoform X1	0.876
Uncharacterized protein LOC101860468	0.821	Laminin subunit β-1	0.707
PDZ and LIM domain protein 7	0.81	Coiled-coil domain-containing protein 18 isoform X1	0.684
Glycogen synthase kinase-3 β	0.758	40S ribosomal protein S8	0.67
Fructose-bisphosphate aldolase	0.721	60S ribosomal protein L8	0.642
T-complex protein 1 subunit epsilon	0.714	60S ribosomal protein L44	0.458
Collagen α 5 (IV) chain	0.703	Ubiquitin	0.379
40S ribosomal protein S8	0.675		–
Ras-related protein Ral-A isoform X1	0.64		–
Aerolysin	0.637		–
60S ribosomal protein L44	0.527		–
60S ribosomal protein L8	0.522		–
Ankyrin repeat domain-containing protein 50, partial	0.357		–
Ubiquitin	0.248		–

These changes in ApKHC1 complex composition could have multiple sources – they could indicate the formation of more ApKHC1 complexes, they could be a product of alterations within the same complex, they could represent a combination of both or due to their regulated release to the synaptic end. Upon normalization by IgG, no significant differences were noted in KHC levels between LTS and STS. To address this uncertainty, and to confirm whether there were any differences in identified cargos to ApKHC1 ratio on induction of STS and LTS, we normalized the dataset with ApKHC1 abundance in the co-IP. The normalization also showed enrichment of different sets of proteins after STS and LTS, with cofilin protein enriched on both types of training (Fig. 2*D*–*F*, Extended Data Fig. 2-1, tables 1–6). Furthermore, analysis of global changes suggested that an uncharacterized protein LOC101861750 commonly depleted after behavioral training (Extended Data Fig. 2-2). These results suggest that STS and LTS induce changes both in the number of ApKHC1 complexes and in their cargo composition.

Based on our previous studies (Liu et al., 2014), we observed that the IP efficiency of samples, as well as the stability of specific proteins interacting with kinesin cargos, can influence the abundance of proteins in individual IP biological replicates. Thus, we further analyzed the dataset to identify protein cargos with synaptic importance—those that were 1.3-fold enriched in the ApKHC1 IPs compared with the control IPs—believing this would help us screen candidates for the WB validation, which is otherwise limited because of the reactivity of commercially available antibodies for *Aplysia* (Extended Data Fig. 3-1, tables 1–4). For further studies, we selected two candidate protein cargos from STS: FKBP2, an endoplasmic reticulum (ER) chaperon, and calumenin, a calcium-binding protein mainly involved in ER functions in protein folding and sorting. Three protein candidates selected from LTS are synaptotagmin-1, dynamin-1, and calmodulin (Extended Data Fig. 3-1, tables 1–4). These candidates were enriched on STS and LTS, respectively, and used for the validation of mass spectrometry data by WB as co-IP in ApKHC1 IPs.

**Figure 3.**
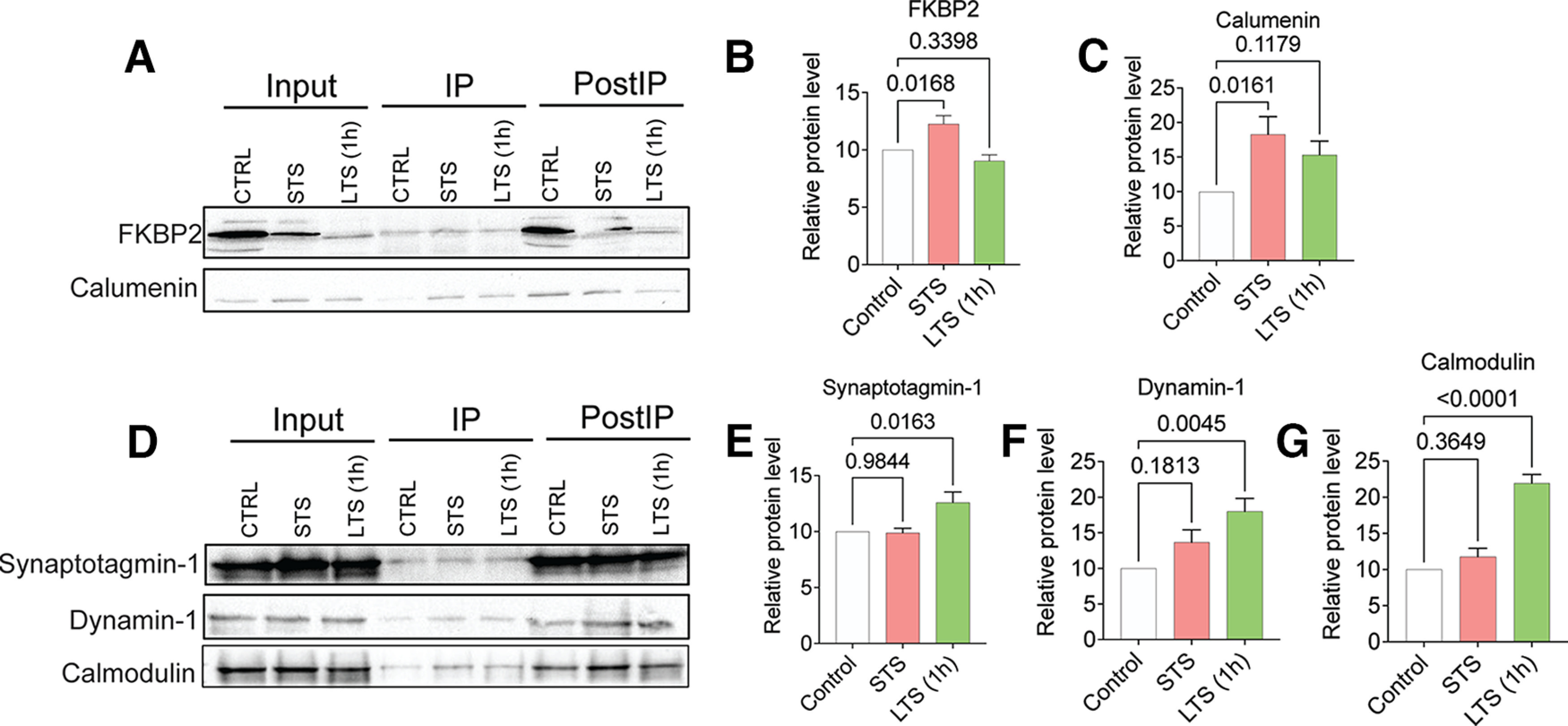
Co-IP and WB validation of quantitative proteomic analysis of ApKHC1 cargos after STS and LTS (1 h). Protein candidates FKBP2 and calumenin selectively enriched on STS, and synaptotagmin-1, dynamin-1, and calmodulin enriched after 1 h of LTS identified by the mass spectrometry analysis were validated in ApKHC1 co-IP by Western blotting. ***A–C***, Validation of FKBP2 and calumenin enrichment in kinesin complex after STS training. ***D–G***, Synaptotagmin-1, dynamin-1, and calmodulin, detected to be enriched in kinesin complex after LTS (1 h) training, are validated by WB. Relative protein levels are expressed as the mean fold change, with error bars showing the SEM. Statistical analyses were conducted by one-way ANOVA followed by Dunnett’s *post hoc* test (Extended Data Fig. 3-1, tables 1–4, Extended Data Fig. 3-2).

10.1523/ENEURO.0266-22.2022.f3-1 Figure 3-1 Table 1, Raw IgG2-normalized data: comparison of detected peptides Control versus STS; 1.3-fold change. Table 2, Raw IgG2-normalized data: comparison of detected peptides Control versus LTS (1 h); 1.3-fold change. Table 3, Selected candidates for Western blot validation experiment. Table 4, WB analysis of candidates selectively enriched in STS and LTS (1 h). Download Figure 3-1, XLS file.

As shown in Figure 3*A*, WB analyses revealed that FKBP2 and calumenin were significantly enriched as co-IP in ApKHC1 IPs after STS. FKBP2 and calumenin levels showed 1.2-fold (Fig. 3*B*; *N* = 5, *p *<* *0.05) and 1.8-fold (Fig. 3*C*; *N* = 5, *p *<* *0.05) increased enrichment compared with control, respectively. FKBP2 and calumenin levels in LTS IPs showed no significant difference (*p *≤* *0.05) compared with control IPs in LTS samples. Examination of synaptotagmin-1, dynamin-1, and calmodulin levels in the WB (Fig. 3*D*), however, found that these synaptic proteins had been significantly enriched in ApKHC1 IPs in response to LTS. Synaptotagmin-1, dynamin-1, and calmodulin levels showed 1.2-fold (Fig. 3*E*; *N* = 5, *p *<* *0.05), 1.7-fold (Fig. 3*F*; *N* = 5, *p *<* *0.01), and 2.1-fold (Fig. 3*G*; *N* = 5, *p *<* *0.001) higher enrichment compared with control IPs (for further details, please see Extended Data Fig. 3-1, tables 1–4, and Extended Data Fig. 3-2). Moreover, no significant differences in enrichment were observed for these three cargo proteins in STS samples compared with controls. These findings indicate that enrichment levels of specific proteins in kinesin complexes are influenced by synaptic signaling and plasticity, as demonstrated by the two sensitization training methods used here.

10.1523/ENEURO.0266-22.2022.f3-2 Figure 3-2 Western blots used to measure the band intensities to calculate the relative protein levels of candidates selected from STS and LTS (1 h) training. ***A***, ***B***, FKBP2 (***A***) and Calumenin (***B***) are enriched upon STS training. ***C–E***, Synaptotagmin-1 (***C***), dynamin-1 (***D***), and calmodulin (***E***) are enriched in LTS (1 h) training. Download Figure 3-2, TIF file.

### TMT-quantitative mass spectrometry of ApKHC1 complexes after 3 h of LTS establishes temporal changes in the composition of the anterograde cargo complex

As described above, different sets of proteins are enriched in the ApKHC1 cargo complex as a response to STS versus LTS. Mechanistically, LTS strengthens existing synaptic connections and leads to the formation of new synapses. One hour after LTS training, we found enrichment of specific proteins functioning in synapse formation. This finding piqued our interest in whether the composition of the ApKHC1 complex could change with additional time elapsed. We wondered whether the kinesin complex transported an entirely different set of proteins to its final destination at the synapses, or whether the final set remained essentially the same. Since the 3–6 h window after LTS is crucial for the facilitation of synaptic connections between the sensory and motor neurons of the siphon- and gill-withdrawal reflex (Ghirardi et al., 1995; Bailey and Kandel, 2008), we assessed the ApKHC1 cargos after 3 h of LTS training.

**Figure 4.**
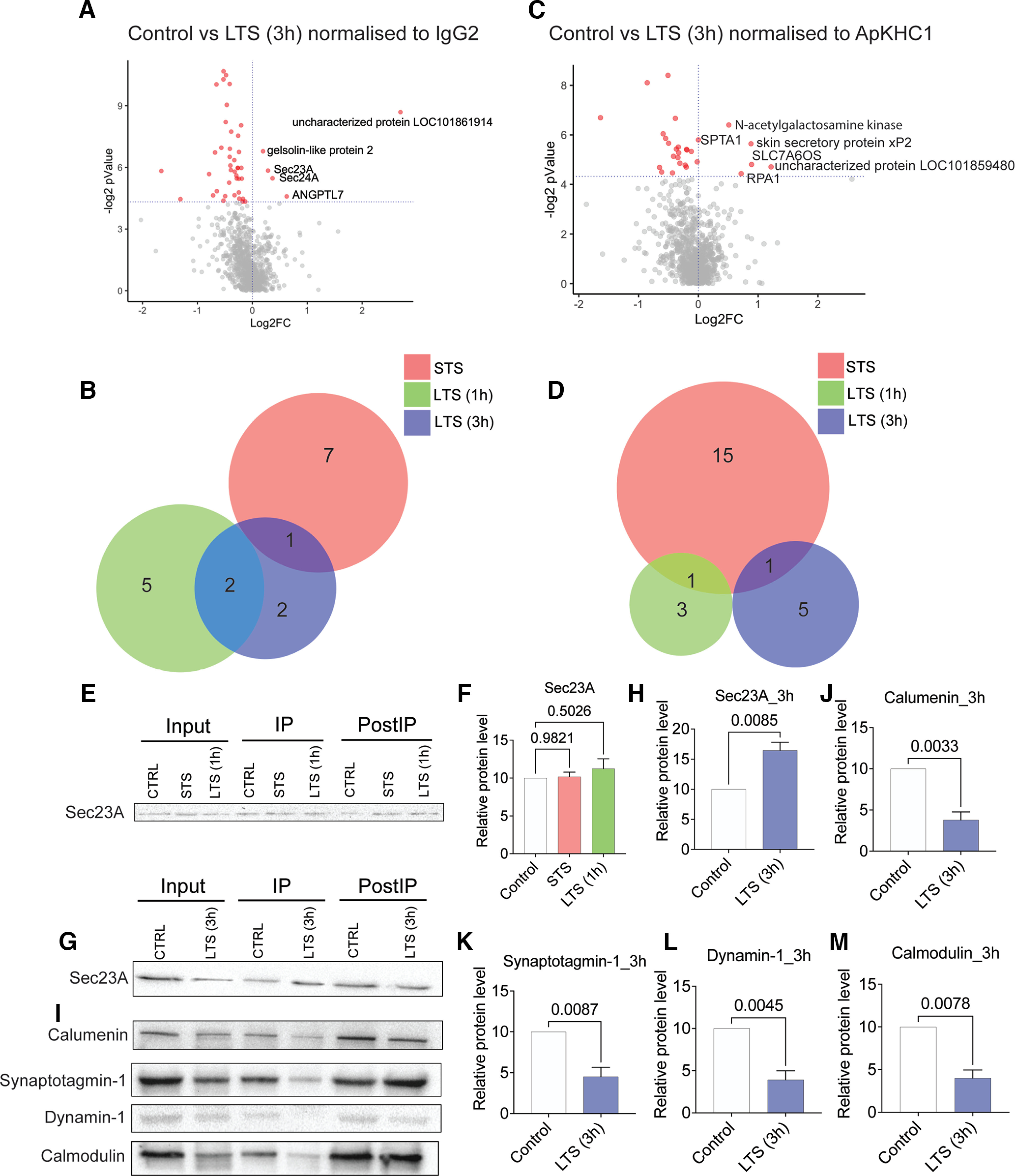
Evaluation of kinesin complexes after 3 h of LTS by mass spectrometry provided evidence of temporal alteration of anterograde cargo complex. Proteins are ranked in a volcano plot according to their statistical -log2 *p*-value (*y*-axis), and their relative abundance ratio (log2 fold change) between higher enrichment and depletion (*x*-axis). Red dots indicate significantly regulated proteins (false discovery rate, <0.01; s0 = 1). ***A***, Volcano plot of control versus LTS (3 h) protein enrichment normalized to IgG2. ***B***, Venn diagram showing the comparison of sets of proteins showed higher enrichment in response to STS, LTS (1 h), and LTS (3 h) normalized to IgG2 (*p *<* *0.05). ***C***, Volcano plot of control versus LTS (3 h) protein enrichment normalized to ApKHC1. ***D***, Venn diagram showing the comparison of sets of proteins showed higher enrichment in response to STS, LTS (1 h), and LTS (3 h) normalized to ApKHC1 (*p *<* *0.05). ***E–M***, ApKHC1 co-IP Western blot validation of proteins enriched in ApKHC1 complex detected by mass spectrometry. ***E***, ***F***, Evaluation of Sec23A enrichment in STS or LTS (1 h) ApKHC1 co-IP by WB. ***G***, ***H***, Sec23A enrichment after 3 h of LTS was confirmed by WB. ***I–M***, Calumenin, synaptotagmin-1, dynamin-1, and calmodulin enrichments were also evaluated in ApKHC1 complexes from LTS (3 h) animals. Relative protein levels are expressed as the mean fold change, with error bars showing SEM; statistical analyses were performed by one-way ANOVA followed by Dunnett’s *post hoc* test and two-tailed paired *t* test (Extended Data Figs. 4-1, 4-2).

To do this, we conducted TMT labeling and LC-MS/MS analysis of the ApKHC1 complexes that we coimmunoprecipitated 3 h after LTS training, as described above. We normalized the dataset with IgG2 and conducted the quantitative comparison of the enriched proteins with the control. Following similar statistical parameters, differential analysis (Fig. 4*A*, Extended Data Fig. 4-1, tables 1–3) revealed that angiopoietin-related protein 7 (ANGPTL7; XP_012938614.1), protein transport protein Sec24A (sec24A; XP_012934788.1), protein transport protein Sec23A (sec23A; XP_012936590.1), gelsolin-like protein 2 (XP_005100380.1), and the uncharacterized protein LOC101861914 (XP_005109882.2) are enriched in the kinesin complexes. We observed that, along with the presence of sec23A and gelsolin-like protein 2, uncharacterized protein LOC101861914 showed ongoing 14.32-fold enrichment after 3 h of LTS (Fig. 4*B*), maintaining the enrichment that it had initially established during STS. Protein transport protein sec24A and ANGPTL7, enriched after 1 h of LTS, also remained after 3 h—a notable finding given that a comparison of global changes suggested the depletion of a number of proteins in the kinesin complex after 3 h of LTS (Extended Data Fig. 4-2). Normalization of the quantitative dataset with ApKHC1 (Fig. 4*C*, Extended Data Fig. 4-1, tables 1–3) showed enrichment of different sets of proteins after 1 and 3 h of LTS (Fig. 4*D*), with only skin secretory protein xP2 isoform enriched in both STS and 3 h after LTS training. In sum, the analysis of global changes, in accordance with the IgG2-normalized dataset, indicated depletion of a number of proteins in the anterograde complex but displayed persistent of others (Extended Data Fig. 4-2).

10.1523/ENEURO.0266-22.2022.f4-1 Figure 4-1 Table 1, Raw IgG2-normalized data: comparison of detected peptides Control versus LTS (3 h). Table 2, Raw kinesin heavy chain 1-normalized data: comparison of detected peptides Control versus LTS (3 h). Table 3, WB analysis of candidates selectively enriched after 3 h of LTS. Download Figure 4-1, XLS file.

10.1523/ENEURO.0266-22.2022.f4-2 Figure 4-2 ***A***, ***B***, Venn diagrams comparing the total number of proteins differentially enriched in ApKHC1 cargos in samples of STS, LTS (1 h), and LTS (3 h). ***A***, Dataset normalized to IgG2. ***B***, Dataset normalized to ApKHC1. ***C–H***, Western blots used to measure the band intensities to calculate the relative protein levels from candidates selected after 3 h of LTS induction. ***C***, Sec23A enriched after 3 h of LTS and showed no significant difference in enrichment after STS and LTS (1 h). ***D***, However, significant enrichment of sec23A is observed after 3 h of LTS. ***E–H***, Calumenin (***E***), synaptotagmin-1 (***F***), calmodulin (***G***), and dynamin-1 (***H***) levels were observed to be significantly depleted after 3 h of LTS. Download Figure 4-2, TIF file.

For the WB validation experiments, we chose sec23A as our candidate for the 3 h time point. As shown in Figure 4*E*, consistent with the proteomics data, we found no significant change in sec23A levels in ApKHC1 complexes from STS and 1 h after LTS training (Fig. 4*F*). However, 1.6-fold higher enrichment (*p *<* *0.01) of sec23A was observed after 3 h of LTS (Fig. 4*G*,*H*). We also examined the levels of previously evaluated candidates at the 3 h time point (Fig. 4*I*–*M*; Extended Data Fig. 4-1, tables 1–3, Extended Data Fig. 4-2). Calumenin, synaptotagmin-1, dynamin-1, and calmodulin levels were ∼54% (*p *<* *0.01), ∼45% (*p *<* *0.01), ∼70% (*p *<* *0.05), and ∼60% (*p *<* *0.01) reduced, respectively, in the kinesin cargos after 3 h of LTS. Based on these findings, we can conclude that LTS induces temporal regulation and synaptic transport in anterograde cargo complexes.

### qRT-PCR analysis of the candidates suggested translational regulation of anterograde cargo loading

**Figure 5.**
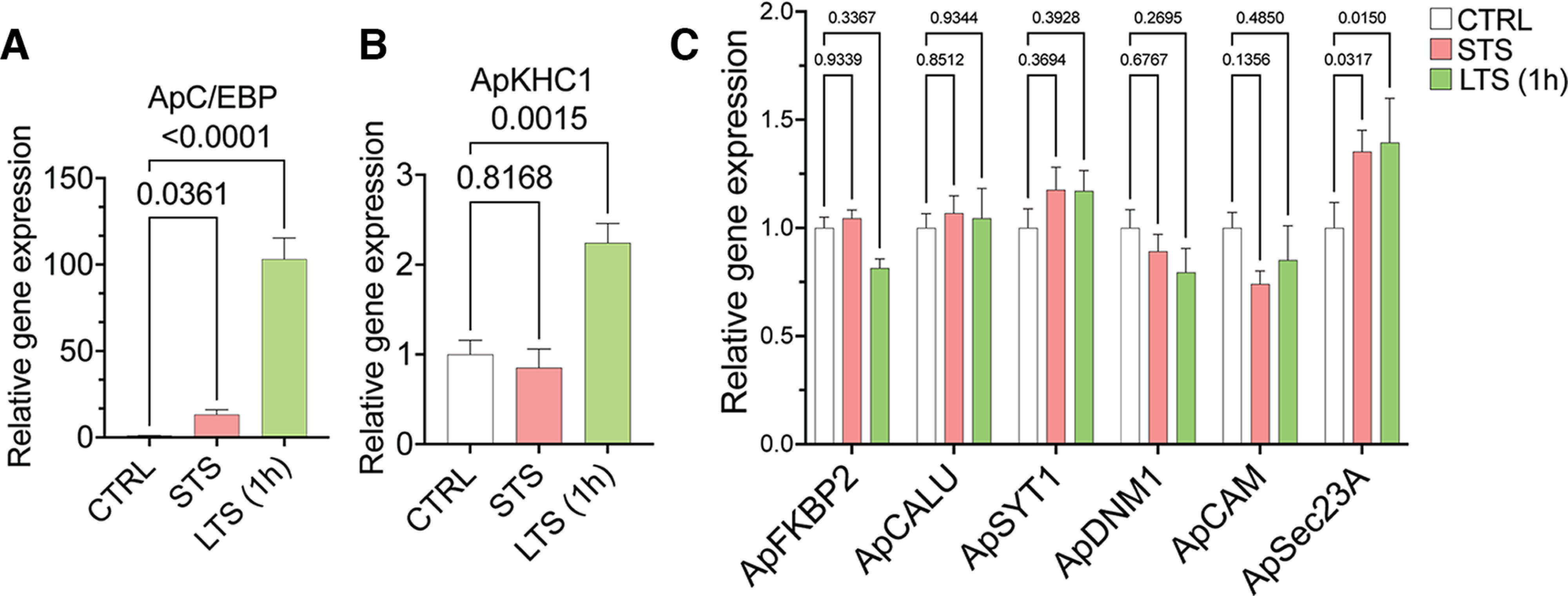
Gene expression analysis by quantitative real-time PCR from sensitized animals. After 1 h of STS and LTS training, RNA was isolated from the entire CNS of the animals (*N* = 5; *n* = 2). ***A***, ***B***, Relative gene expression analysis of ApC/EBP (***A***) and ApKHC1 (***B***) in STS and LTS (1 h) animals. ***C***, Relative gene expression profile of FKBP2, calumenin, synaptotagmin-1, dynamin-1, calmodulin, and sec23A in response to STS and LTS (1 h). Relative gene expression levels are expressed as the mean fold change, with error bars showing SEM; statistical analyses were performed by one-way ANOVA followed by Dunnett’s *post hoc* test (Extended Data Fig. 5-1).

Since the proteomics analyses showed differential cargo enrichment in anterograde complex on STS or LTS, we wanted to check whether these proteins were regulated at their transcript level. Thus, we studied the expression level of the candidates found highly enriched in the anterograde complex, namely FK506-binding protein 2 (ApFKBP2; XM_005099915.3), calumenin (ApCALU; XM_005110250.3), synaptotagmin-1 (ApSYT1; NM_001204624.1), dynamin-1 (ApDNM1; XM_005106526.3), calmodulin (ApCAM; XM_005095332.3); and ApSEC23A (XM_013081136.2), on induction of STS and LTS. A list of primers used in the study has been provided in Extended Data Figure 5-1 (tables 1, 2). We used Aplysia CCAAT enhancer-binding protein (ApC/EBP), an immediate-early gene, which is essential for the successful consolidation of long-term memory, and plasticity as the positive control (Alberini et al., 1994; Puthanveettil et al., 2013). As reported previously by Puthanveettil et al. (2008), an expected rise in ApC/EBP (∼93-fold increase over control; *p *<* *0.001) and ApKHC1 (greater than twofold increase over control; *p *<* *0.01) levels were found on LTS, which validates the induction of LTF in the LTS samples (Fig. 5*A*,*B*). Relative gene expression values (with *p*-values) are provided in Extended Data Figure 5-1 (tables 1, 2). Other than ApSEC23A, all analyzed candidates showed no significant alteration in gene expression on STS and LTS (1 h) training (Fig. 5*C*). This suggests that at that time point, the proteins are being regulated at their translational level or some factor that controls/selects the protein needs to be transported from the cell body based on the synaptic need. In our proteomics experiment, we observed ApSec23A enrichment after 3 h of LTS. This could suggest that the upregulation of ApSec23A gene expression after 1 h of LTS reflected protein enrichment after 3 h in ApKHC1 cargos. Given these observations, combining the proteomics study with the gene expression analysis, we can assume that most of the regulations of cargo loading onto the anterograde complex could take place at protein level, and the cargo is selected based on the designated activity.

10.1523/ENEURO.0266-22.2022.f5-1 Figure 5-1 Table 1, Primers used for qRT-PCR analysis. Table 2, Relative gene expression analysis by qRT-PCR. Download Figure 5-1, XLS file.

## Discussion

LTM formation is an intricately complex process. It is represented by the seamless orchestration of signal transduction, transcriptional regulation, and protein synthesis, resulting in a structural change to the neural circuitry (Kandel, 2001; Bliss et al., 2003). Physiologic regulation of soma-to-synapse communication mediated by KIFs serves as a critical component of this synaptic and structural plasticity (Puthanveettil et al., 2008; Swarnkar et al., 2021). Using the well characterized siphon withdrawal reflex of *Aplysia*, which facilitates robust behavioral plasticity and nonassociative learning-induced LTM in regard to both short-term and long-term sensitization (Pinsker et al., 1970, 1973; Carew et al., 1971, 1981; Kandel, 2001), we assessed whether KIFs mediate the transport of specific proteins during learning. By quantitative proteomic analyses in this study, we explored the role of kinesins further by discovering that different sets of proteins are enriched in the anterograde cargo complex ApKHC1 in response to learning.

Our training protocol for inducing LTS involved four bouts of electric shocks separated by 20 min to the posterior of the body wall (Pinsker et al., 1970, 1973; Carew et al., 1971, 1981; Kandel, 2001), taking a total of 2 h for stimulation. We then isolated the CNS 1 or 3 h after training for ApKHC1 complex immunoprecipitation. However, an intermediate form of sensitization [intermediate-term sensitization (ITS)], which lasts 1–3 h after training, could be produced by our stimulation as well. ITS could be induced by spaced tail shocks in 15 min intervals. ITS lasts ∼90 min post-training and decays completely after 3 h (Ghirardi et al., 1995; Mauelshagen et al., 1996; Sutton and Carew, 2000; Sutton et al., 2001, 2002, 2004). In our sensitization experiments, gene expression analysis showed a significant increase in ApC/EBP (an immediate early gene) levels, in the LTS (1 h) group. Since intermediate forms of memory require only new protein synthesis, but not gene expression (Sutton et al., 2001), our LTS (1 h) data, which are from 3 h after the first tail shock, reflect an early phase of LTM (overlapping with ITS), whereas the LTS (3 h) data represent specific changes related to LTM.

### ApKHC1 complex is enriched with proteins involved in synaptic structural plasticity

TMT labeling (Tuli and Ressom, 2009; Zhang and Liu, 2017) enabled our ability to detect and quantify small amounts of peptides associated with ApKHC1 on sensitization, and on a database search of the trypsinized peptides we could identify >1000 protein IDs. This includes previously identified kinesin cargos such as synaptic vesicle precursors synaptotagmins, rab3 (Okada et al., 1995; Niwa et al., 2008; McVicker et al., 2016), synaptic membrane precursor syntaxin-binding protein 1 (Su et al., 2004), AMPA receptor subunit (Setou et al., 2002), mitochondrial proteins (Nangaku et al., 1994; Cai et al., 2005; Wozniak et al., 2005; Glater et al., 2006; Cho et al., 2007; Wang and Schwarz, 2009), tubulin (Kimura et al., 2005), neural cell adhesion molecule (Wobst et al., 2015), components of proteasome complex (Otero et al., 2014), RNA-binding proteins, hnRNPs, and a putative cargo adaptor tropomyosin (Chennathukuzhi et al., 2003; Kanai et al., 2004; Wu et al., 2020; Dimitrova-Paternoga et al., 2021). We could also identify multiple proteins that had been recognized as synaptic cargo in our previous study (Liu et al., 2014). Additionally, we identified several other proteins throughout the course of this project, such as different ribosomal proteins that aid in local translations at synaptic ends (Fusco et al., 2021; Dastidar and Nair, 2022), angiopoietins and flotillin that support neurite outgrowth and synaptogenesis (Kosacka et al., 2006; Swanwick et al., 2010), eukaryotic translation initiation factors (Swarnkar et al., 2021), RNA-binding proteins (Kanai et al., 2004), calcium-binding proteins and calcium/calmodulin-dependent kinases, cAMP-dependent protein kinase regulatory subunit, catalytic subunit of protein kinase A, PKA type II regulatory subunit, MAPK1, and multiple ras-related proteins that play crucial roles in synaptic facilitation, signaling, and memory formation (Castellucci et al., 1980; Kandel, 2001; Ye and Carew, 2010; Lisman et al., 2012; Monje et al., 2012). Other than the proteins with synaptic importance, these cargos transported by ApKHC1 revealed proteins that carry out fundamental neuronal functions as well as proteins involved in neuronal disease manifestation. For example, highly conserved multifunctional 14-3-3 proteins (Cornell and Toyo-oka, 2017) complement protein C1q (Benoit and Tenner, 2011), essentially participating in neural development and neuroprotection. 7B2 precursor (Jarvela et al., 2018), cathepsin B (Hook et al., 2020), and heat shock protein 70 (Turturici et al., 2011) were detected as ApKHC1 cargo is involved in Parkinson’s disease, Alzheimer’s disease, polyglutamine diseases, and amyotrophic lateral sclerosis. Therefore, the present study validates the synaptic mechanics as well as the cellular significance of kinesin motor protein in directional intracellular transport in response to learning-induced memory formation.

We tried to carry out Gene Ontology analysis with the identified protein IDs associated with kinesin cargos, however, because of the lack of well defined available functional GO term annotations of *Aplysia* genome, we could not run the analysis for biological processes, molecular functions, and cellular components. Based on the STRINGdb 11.5 (string-db.org) and using the ShinyGO 0.76.2 online tool, we did network analysis of the protein domains (Pfam; Extended Data Fig. 1-2). RNA recognition motif, Ras family, and EF-hand domains are some of the nodes observed that are directly associated with neuronal functions as described in the previous paragraph.

### Activity-dependent alterations in ApKHC1 cargos after STS and LTS training

The quantitative comparison of ApKHC1-transported cargos, analyzed from STS-trained and LTS-trained *Aplysia* showed activity-dependent differential enrichment and depletion of proteins in the anterograde cargo. For the co-IP, we used an equal amount of anti-ApKHC1 antibody, so we normalized the dataset to IgG2 to equilibrate any unwanted (nonbiological) variations among the samples and to achieve an accurate statistical assessment.

Among the proteins enriched on STS, we identified those with a fundamental role in performing a basal cellular function, such as Hsc70, DDX3Y in neural development (Vakilian et al., 2015); ERH in pyrimidine metabolism, DNA replication, and cell proliferation (Fujimura et al., 2012); and FKBP2, which processes peptidyl-prolyl *cis*/*trans* isomerase (PPIase) domains (Tong and Jiang, 2015), a subunit of the V1 complex of vacuolar(H+)-ATPase (V-ATPase)-ATP6V1C1. We also found the following RNA-binding proteins: hnRNP1, which participates in mRNA transport and protein translation (Clarke et al., 2021) as well as CELF1 functioning in RNA processing (Gallo and Spickett, 2010; Ladd, 2013). On the other hand, after 1 h of LTS, enrichment of synaptotagmin-1 along with dynamin-1 and other Ca^2+^-sensing proteins, NECAB1 and calmodulin, suggests that, as a result of LTS, kinesin is transporting proteins participating in synapse strengthening and memory formation to the neuronal ends. Synaptotagmin-1, a presynaptic vesicle protein interacts with neurexin and SNAP-25, and is essential as a Ca^2+^ sensor for fast and synchronous synaptic vesicle fusion (Monje et al., 2012; Chang et al., 2018), whereas dynamin-1 is critically required for synaptic vesicle endocytosis (Raimondi et al., 2011; Fa et al., 2014). Calmodulin is required for the activation of CAMKII (Mayford et al., 1996; Murakoshi et al., 2017). In *Aplysia*, synaptotagmin-1 has been reported to have an inhibitory effect on synaptic vesicle release (Martin et al., 1995). Nonetheless, our findings are in agreement with those of the study by Monje et al. (2012), in which enhanced levels of synaptotagmin-1 are present in *Aplysia* cerebral ganglia after 24 and 48 h of serotonin treatment. Intriguingly, we could find only some of the components of synaptic vesicle proteins. While cargos of kinesins consists or multiprotein complexes, organelles, and vesicles, the precise mechanisms by which they transport these cargos remains to be understood in detail. One would reasonably expect to identify all the components of these cargos, probably because of the co-IP conditions, which include the use of mild detergents, we identified only some of their components.

Enrichment of F-actin barbed-end-capping and severing protein gelsolin-like protein 2 (Burtnick et al., 1997) after 3 h of LTS suggests that after establishing new synaptic connections, actin filaments at the end of the neurites of the newly formed synaptic ends might be stabilized for the maintenance of newly formed memory (Bailey et al., 2008). Sensitization also showed depletion of the components of multiprotein complexes such as ribosomal complex among the protein cargos following both STS and LTS. STS led to 21.4%, LTS (1 h) led to 42.85%, and LTS (3 h) led to 40% depletion of the ribosomal proteins of the total depleted proteins (according to the *p *<* *0.05, IgG2-normalized data). Reduced association of these proteins with ApKHC1 in response to STS/LTS training further confirms that sensitization training alters the composition of protein cargos transported by ApKHC1, including those that are the integral components of protein translation and degradation machineries. Furthermore, the proteins that show decreased association with ApKHC1 on training could be regulated in different ways, for example by local translation or transport by other motor proteins. Together, these results further confirm that kinesin-mediated transport and its cargos are targets of regulation during learning and LTM.

A conserved domain search of LOC101861914, which is noted to be highly enriched on STS and 3 h after LTS, has shown that the uncharacterized protein contains EF hand motifs, with helix-loop-helix structural domain predominantly observed in Ca^2+^-binding proteins. Using an online tool at www.swissmodel.expasy.org, structural prediction of the amino acid sequence showed homology with other Ca^2+^-interacting proteins calcium binding domain of CpCDPK3, calexcitin, CDPK1, and calcyphosin. Crystal structure and biochemical characterization of recombinant human calcyphosin delineates a novel EF hand-containing protein family that shows 21.43% sequence identity (Extended Data Fig. 1-2) with uncharacterized protein LOC101861914. Thus, the current study uncovers a putative calcium-binding protein that is significantly enriched in the anterograde complex on sensitization; however, detailed characterization is required to discover its role during synaptic plasticity.

We also observed enrichment of protein transport proteins sec23A/sec24A after 3 h of LTS. Sec23/24 heterodimer is an integral component of the COPII coat complex that forms on the endoplasmic reticulum and transports cargo from the endoplasmic reticulum to Golgi (Bi et al., 2002; Lord et al., 2013). Furthermore, coordinated local protein synthesis and degradation are essential for the maintenance of homeostatic plasticity (Cajigas et al., 2010), and we assume that sec23/24 protein delivery plays a key role in this plasticity by transporting vesicles from ER to Golgi after 3 h of LTS in newly formed, stabilized neural ends. Since the function of gelsolin-like proteins and sec23/24 have not yet been studied extensively in learning and memory, these results serve as a platform for further exploration. We assume that after LTS—based on the changing temporal needs of neurons in the ApKHC1 complex, and after the establishment and remodeling of new and existing synaptic connections—the system might be moving toward homeostasis. Thus, these findings suggest that protein loading in kinesin cargos happens in an activity-dependent manner and that there might be a motor-cargo code determining the specificity of anterograde transport of a distinct cargo.

We also found that after LTS training, unlike STS, there was a significant increase in ApKHC1 protein expression. Based on our qRT-PCR analysis, we observed that cargos loaded onto ApKCH1 are regulated mostly at the protein/translational level. Therefore, the question is then raised as to whether the changes in ApKHC1 complex composition could be because of the formation of more ApKHC1 complexes, the loading of more proteins into the same complex, or simply the combined effects of both. Therefore, we further analyzed the dataset after normalizing it with ApKHC1 to check whether more or fewer cargos were being loaded onto the ApKHC1 complex. This analysis showed that following STS, a significantly higher number of proteins was enriched in the ApKHC1 complex compared with 1 h after LTS. Since STS did not alter ApKHC1 levels, these results suggest an increase in the number of enriched proteins in the ApKHC1 complex during STS, whereas LTS might involve both an increase in the number of ApKHC1 complexes as well as the specific enrichment of proteins within the same complex.

**Figure 6.**
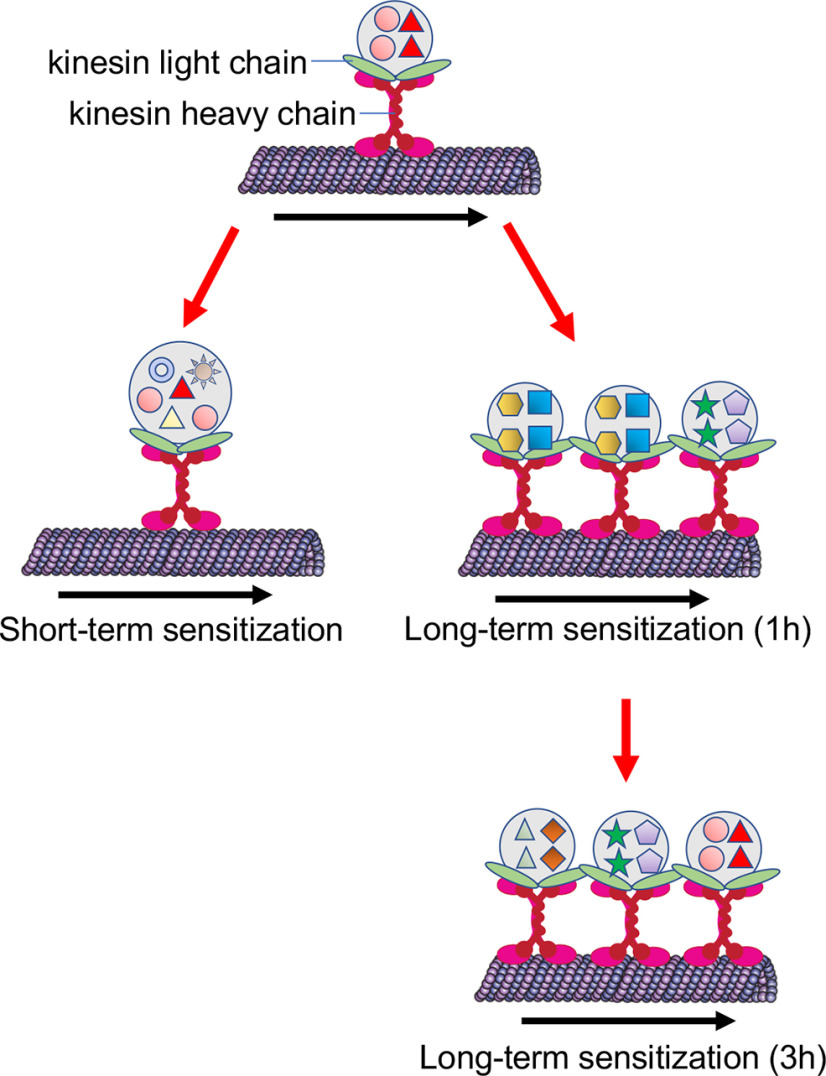
In response to LTS, ApKHC1 gets upregulated and increases anterograde transport, but the ApKHC1 level remains unaltered in response to STS. Results from the current investigation showed that on STS training, protein enrichment into each ApKHC1 complex increased significantly compared with LTS. However, in response to LTS, selective enrichment of proteins occurs within the same complex along with the increase in the number of ApKHC1 complex formations.

Our studies establish that both the composition and number of ApKHC1 complexes are targets of regulation during learning (Fig. 6). These dynamic changes in ApKHC1 complexes facilitate soma-to-synapse communication. They also reflect adjustments in the synaptic proteome that facilitate structural changes and synaptic plasticity during learning. Identification and characterization of circuit-specific KIF complexes will help bring deeper insights into the regulation of the communication between soma and synapse during LTM.

## Synthesis

Reviewing Editor: Katalin Toth, University of Ottawa Decisions are customarily a result of the Reviewing Editor and the peer reviewers coming together and discussing their recommendations until a consensus is reached. When revisions are invited, a fact-based synthesis statement explaining their decision and outlining what is needed to prepare a revision will be listed below. The following reviewer(s) agreed to reveal their identity: Wayne Sossin. Note: If this manuscript was transferred from JNeurosci and a decision was made to accept the manuscript without peer review, a brief statement to this effect will instead be what is listed below.

Both reviewers found the manuscript interesting and the presented data valuable that advances the field. However, both identified several points where the study should be strengthened. Their detailed points are listed below, these should be carefully addressed by the authors.

Rev. #1.

The study compares proteomics of kinesin Ips between control and sensitized animals using the Aplysia model system. Results are accomplished 1h after short-term sensitization, and for 1 and 3h after long-term sensitization. Certain proteins are confirmed by immunoblotting and whether or not gene expression changes (using qRT-PCR are done). In conclusion several proteins are identified whose association with kinesins change during both short and long-term sensitization. This is an interesting study that is well controlled and adds to our information on changes that occur during the process of memory formation. However, there are several issues with how the data is quantified (normalization) and interpreted that need to be addressed. There are also a number of other issues where more clarity is required

Major Issues

1. Issues with normalization.

Line 308-311.

“These changes in ApKHC1 complex composition could have multiple sources - they could indicate the formation of more ApKHC1 complexes, they could be a product of alterations within the same complex, or they could represent a combination of both. To address this uncertainty, we normalized the data with ApKHC1 abundance in the co-IP. ”

This is partly true, although it could also be due to changes in which type of cargo is on kinesin, either because more are formed, or more are released. However, is not clear to me how normalizing for kinesin vs normalizing for Igg addresses why the ApKHC1 complex composition changes and the two different normalizations give very different results (see point below). This logic needs to be explained much more clearly here.

There appear to have been two TMT experiments to give an overall n of 5 for the four groups. The TMT is internally controlled allowing for any peptide to be compared between the groups. However, it is true that there is variability within an IP and if there is considerable differences in the amount of IGG seen, it could be reasonable to use this for normalization, although it is not clear that this should be necessary. How big a normalization was it? (i.e. how difference was the Igg levels or kinesin levels from different Ips?). Also, since kinesin levels increase in LTS, but not STS, is this also seen in the Ips?

In particular, it is disturbing that for at least one protein (the LOC101861914), it is significantly increased (14.32) when normalizing to Igg, but significantly decreased (0.925) when normalized to Kinesin); both for STS. I don’t understand how this can be right.

2. Not enough attention to decreases in association with Kinesin

While mentioned, not enough attention is given to the significant decreases in association with kinesin. In particular, it appears that ribosomal proteins and to some extent RNA binding proteins are enriched in the proteins associated with kinesin. Since RNA granules may be released during learning, it seems as if this should be more carefully looked at.

Moreover, while changes in individual ribosomal subunits may be significant or not, since the small subunit and the large subunit are for the most part a large complex, and since kinesin is known to transport an RNA granule containing ribosomes/polysomes, the overall enrichment and de=enrichment of the small and large subunit (i.e. the average change for all ribosomal subunits) should be presented. A decrease could be interpreted as the break-up of the RNA granule which has been reported for various forms of plasticity in other systems.

3. Looking at kinesin cargo in a more organized manner. Kinesin does not transport individual proteins, but vesicles/organelles. For example, synaptotagmin is transported by kinesin as synaptic vesicle precursors/synaptic vesicles. There are many other known proteins in these structures. Addressing whether they all change or just one protein changes addresses important issues as to what may be occurring with transport. No attempt is made at addressing organelle transport using known components in the proteomic analysis and I believe this would be useful. Similarly attention is paid to Sec 23, part of the CopII complex. Do other members of Cop II show similar changes as Sec23?

Minor comments

Line 151: I assume this is micrograms of protein, not milligrams of protein loaded on the gel.

Tale S5; it is not clear why some of the proteins are shaded in yellow. Are these the ones with synaptic localization and function? What criteria were used for determining this?

Instead of listing the proteins on lines 289-302. This major result should really involve taking the part of the supplemental table and making it a table in the main part of the paper. Both the upregulated and downregulated proteins should be shown in separate tables.

There is too little description of the function of the proteins examined. The unidentified LOC protein has an EF hand is part of a larger EF hand containing family. This should be discussed.

Lines 397-399 “Therefore, the enrichment of proteins observed after 1h, could be due to the upregulation of the genes at an earlier time point, and no significant change in gene expression contributes to their depletion after three hours. ”

This makes no sense to me. First there is no evidence for upregulation of gene expression at earlier time points. Second, the comparison is to control where there is no change in gene expression, so no significant change in gene expression is the same as the control and cannot explain a difference from control.

I found the discussion to be just a listing of the proteins identified without much insight into the actual findings. The title “ApKHC1-complex is enriched with proteins involved in synaptic structural plasticity” is not at all backed up by the ensuing paragraph. Based on the list, almost any protein could satisfy this term “Involved in synaptic structural plasticity”. Moreover, most of the proteins are downregulated, not upregulated. Indeed, the lack of discussion of downregulated genes is a problem (see major points); the summary figure (6) completely ignores that most of the proteins changed, especially at 3hrs, but also at the other points are decreased in KHC, not increased. .

Lines 486-490: Neitehr Increases in synaptotagmin nor dynamin at synapses would be expected to lead to increases in transmitter release. These may reflect a general increase in the transport of synaptic vesicle components, but the functions of these proteins is not thought to be a rate limiting step in transmitter release and I am not aware of any literature where small changes in the levels of these protein is sufficient to alter release. Indeed, overexpressing synaptotagmin in Aplysia sensory neurons did not lead to changes in transmitter release; if anything, overexpression leads to a decrease in transmitter release 10.1073/pnas.92.24.11307; doi: 10.1523/JNEUROSCI.23-15-06238.200

Lines 514-516. “Also, based on our qPCR analysis, we observed that cargos loaded onto ApKCH1 are regulated mostly at the transcriptional level “

1/6 of the proteins examined had a transcriptional change, this sentence makes no sense. Moreover, the change was seen at 1 hr, but the increase in kinesin association was only seen at 3 hrs.

Rev. #2.

This paper examines the nature of anterograde soma-to-synapse transport of molecules via the Aplysia motor protein kinesin (ApKHC1) during sensitization in Aplysia. Previous work by the senior author and authors has implicated the kinesin family of molecular motor proteins (KIFs) in coordinating soma-to-synapse intracellular communication. Here, the authors investigate how the molecular cargo transported by a specific motor protein kinesin, ApKHC1, changes during various phases of memory following training for sensitization in Aplysia. They analyze the composition of ApKHC1 complexes at following two types of training: a single tail shock, which produces short-term (30 min to 1 hr) sensitization (STS) and four spaced tail shocks which produces long-term ({greater than or equal to} 24 hr) sensitization (LTS). The examined ApKHC1 complexes at 1 hr after one shock (short-term memory [STM]), as well as 1 hr and 3 hr after four shocks (LTM). For the molecular analyses almost the entire CNS (cerebral, pleural, pedal, and abdominal ganglia) was removed from the animals.

Proteomic analyses of the CNS from trained and control animals were performed by co-immunoprecipitation of ApKHC1 complexes followed by labeling of peptides by tandem mass tag (TMT) and subsequent quantification using mass spectroscopy (MS). These analyses showed that STS, as assessed 1 hr after one tail shock, produced enrichment of different sets of proteins in ApKHC1 complexes than did LTS as assessed 1 hr after four tail shocks. Specifically, for LTS after 1 h there was enrichment in the complexes of proteins involved in synaptic function and synapse formation, including synaptotagmin-1 and the neuronal calcium binding protein NECAB1. Differential proteomic analyses of control and sensitized animals 3 hr after four shocks revealed additional changes in ApKHC1 complexes. In particular, LTS, assessed 3 hr after four shocks, showed enrichment for a set of proteins that largely differed from the set for LTS after 1 hr. Such proteins as the protein transport proteins and an actin-binding protein gelsolin-like protein 2 were enriched in ApKHC1 complexes 3 hr after LTS training, but not at 1 hr after LTS training. Other analyses using western blot validation of the MS data showed that after 1 hr LTS, but not STS, enriched ApKHC1 complexes for proteins associated with synaptic function and plasticity, including synaptotagmin-1, dynamin-1, and calmodulin.

The authors also examined whether sensitization altered ApKHC1 cargo at the transcriptional level. Of the cargo proteins that were enriched in either STS or LTS only one candidate, ApSec23A, exhibited increased expression after 1 hr.

The finding of alterations in the cargo of anterograde motor protein kinesin due to learning and, in particular, to specific phases of memory represents a significant advance in the field. It is fascinating that sensitization in Aplysia results in largely distinct ApKHC1 cargos depending on the amount of training (one shock vs. three shocks) and the time after training (1 hr vs. 3 hr). The results generally indicate that 1 hr after sensitization training with four shocks there is prominent ApKHC1-mediated delivery of synaptic proteins to synapses. This finding, in turn, accords well with extensive prior research in Aplysia showing that sensitization is accompanied by synaptic growth.

The results are clearly presented, and the statistical analyses appear valid. Furthermore, the paper is well written. I have only one major comment and a few minor suggestions for improving the manuscript.

Major Comment:

There are no behavioral data showing that the training methods actually resulted in the types of sensitization that the authors claim. Did one tail shock produce enhancement of the siphon-withdrawal reflex that was indeed short-term, that is, that persisted for {less than or equal to} 1 hr? Is it possible that the single shock used in the study produced sensitization that persisted for > 1 hr? If so, then what the authors call STS might instead be intermediate-term sensitization (ITS), and ITS is mechanistically distinct from STS. Also, did four shocks induce true LTS, LTS that lasts for {greater than or equal to} 24 hr?

Minor Comments:

1. Lines 65-67: STM in Aplysia is generally regarding as lasting 30-min-to-1-hr. Sensitization that lasts from 1-3 hr is referred to as intermediate-term memory (ITM). LTM lasts {greater than or equal to} 24 hr. Classically, STM does not require either protein synthesis or gene transcription; ITM requires protein synthesis, but not gene transcription; and LTM requires both protein synthesis and gene transcription. Here, the authors conflate STM and ITM.

2. Line 99: Remove the scare quotes from sensitization.

3. Line 147: thrice is archaic. Instead, use “three times” or “3x”.

4. Lines 163 and 166: “4μg” vs. “35 μL”-be consistent in spacing.

5. Lines 169 and 173: “manufacturer”s instructions” vs. “manufacturer instruction”-be consistent.

## References

[B1] Alberini CM, Ghirardl M, Metz R, Kandel ER (1994) C/EBP is an immediate-early gene required for the consolidation of long-term facilitation in Aplysia. Cell 76:1099–1114. 10.1016/0092-8674(94)90386-7 8137425

[B2] Antonov I, Kandel ER, Hawkins RD (1999) The contribution of facilitation of monosynaptic PSPs to dishabituation and sensitization of the *Aplysia* siphon withdrawal reflex. J Neurosci 19:10438–10450. 10.1523/JNEUROSCI.19-23-10438.1999 10575041 PMC6782414

[B3] Antonov I, Antonova I, Kandel ER, Hawkins RD (2003) Activity-dependent presynaptic facilitation and Hebbian LTP are both required and interact during classical conditioning in Aplysia. Neuron 37:135–147. 10.1016/s0896-6273(02)01129-7 12526779

[B4] Badal KK, Akhmedov K, Lamoureux P, Liu XA, Reich A, Fallahi-Sichani M, Swarnkar S, Miller KE, Puthanveettil SV (2019) Synapse formation activates a transcriptional program for persistent enhancement in the bi-directional transport of mitochondria. Cell Rep 26:507–517.e3. 10.1016/j.celrep.2018.12.073 30650345 PMC6380353

[B5] Bailey CH, Kandel ER (2008) Synaptic remodeling, synaptic growth and the storage of long-term memory in Aplysia. Prog Brain Res 169:179–198. 10.1016/S0079-6123(07)00010-6 18394474

[B6] Bailey CH, Barco A, Hawkins RD, Kandel ER (2008) Molecular studies of learning and memory in *Aplysia* and the hippocampus: comparative analysis of implicit and explicit memory storage. In: Learning and memory: a comprehensive reference (Byrne JH, ed), pp 11–29. Oxford: Elsevier.

[B7] Benoit ME, Tenner AJ (2011) Complement protein C1q-mediated neuroprotection is correlated with regulation of neuronal gene and microRNA expression. J Neurosci 31:3459–3469. 10.1523/JNEUROSCI.3932-10.2011 21368058 PMC3080046

[B8] Bi X, Corpina RA, Goldberg J (2002) Structure of the Sec23/24–Sar1 pre-budding complex of the COPII vesicle coat. Nature 419:271–277. 10.1038/nature01040 12239560

[B9] Bilsland LG, Sahai E, Kelly G, Golding M, Greensmith L, Schiavo G (2010) Deficits in axonal transport precede ALS symptoms in vivo. Proc Natl Acad Sci U S A 107:20523–20528. 10.1073/pnas.1006869107 21059924 PMC2996651

[B10] Bliss TV, Collingridge GL, Morris RG (2003) Introduction. Long-term potentiation and structure of the issue. Philos Trans R Soc Lond B Biol Sci 358:607–611. 10.1098/rstb.2003.1282 12740102 PMC1693168

[B11] Burtnick LD, Koepf EK, Grimes J, Jones EY, Stuart DI, McLaughlin PJ, Robinson RC (1997) The crystal structure of plasma gelsolin: implications for actin severing, capping, and nucleation. Cell 90:661–670. 10.1016/s0092-8674(00)80527-9 9288746

[B12] Byrne JH, Hawkins RD (2015) Nonassociative learning in invertebrates. Cold Spring Harb Perspect Biol 7:a021675. 10.1101/cshperspect.a021675 25722464 PMC4448621

[B13] Cai Q, Gerwin C, Sheng ZH (2005) Syntabulin-mediated anterograde transport of mitochondria along neuronal processes. J Cell Biol 170:959–969. 10.1083/jcb.200506042 16157705 PMC1804288

[B14] Carew TJ, Castellucci VF, Kandel ER (1971) An analysis of dishabituation and sensitization of the gill-withdrawal reflex in Aplysia. Int J Neurosci 2:79–98. 10.3109/00207457109146995 4347410

[B15] Carew TJ, Walters ET, Kandel ER (1981) Classical conditioning in a simple withdrawal reflex in *Aplysia californica*. J Neurosci 1:1426–1437. 10.1523/JNEUROSCI.01-12-01426.1981 7320755 PMC6564124

[B16] Cajigas IJ, Will T, Schuman EM (2010) Protein homeostasis and synaptic plasticity. EMBO J 29:2746–2752. 10.1038/emboj.2010.173 20717144 PMC2924649

[B17] Castellucci VF, Kandel ER, Schwartz JH, Wilson FD, Nairn AC, Greengard P (1980) Intracellular injection of the catalytic subunit of cyclic AMP-dependent protein kinase simulates facilitation of transmitter release underlying behavioral sensitization in Aplysia. Proc Natl Acad Sci U S A 77:7492–7496. 10.1073/pnas.77.12.7492 6111794 PMC350531

[B18] Chang S, Trimbuch T, Rosenmund C (2018) Synaptotagmin-1 drives synchronous Ca2+-triggered fusion by C2B-domain-mediated synaptic-vesicle-membrane attachment. Nat Neurosci 21:33–40. 10.1038/s41593-017-0037-5 29230057 PMC5742540

[B19] Chennathukuzhi V, Morales CR, El-Alfy M, Hecht NB (2003) The kinesin KIF17b and RNA-binding protein TB-RBP transport specific cAMP-responsive element modulator-regulated mRNAs in male germ cells. Proc Natl Acad Sci U S A 100:):15566–15571. 10.1073/pnas.2536695100 14673085 PMC307608

[B20] Cho KI, Cai Y, Yi H, Yeh A, Aslanukov A, Ferreira PA (2007) Association of the kinesin‐binding domain of RanBP2 to KIF5B and KIF5C determines mitochondria localization and function. Traffic 8:1722–1735. 10.1111/j.1600-0854.2007.00647.x 17887960

[B21] Chu Y, Morfini GA, Langhamer LB, He Y, Brady ST, Kordower JH (2012) Alterations in axonal transport motor proteins in sporadic and experimental Parkinson’s disease. Brain 135:2058–2073. 10.1093/brain/aws133 22719003 PMC4571141

[B22] Clarke JP, Thibault PA, Salapa HE, Levin MC (2021) A comprehensive analysis of the role of hnRNP A1 function and dysfunction in the pathogenesis of neurodegenerative disease. Front Mol Biosci 8:659610. 33912591 10.3389/fmolb.2021.659610 PMC8072284

[B23] Cornell B, Toyo-Oka K (2017) 14-3-3 proteins in brain development: neurogenesis, neuronal migration and neuromorphogenesis. Front Mol Neurosci 10:318. 29075177 10.3389/fnmol.2017.00318 PMC5643407

[B24] Cross JA, Woolfson DN, Dodding MP (2021) Kinesin-1 captures RNA cargo in its adaptable coils. Genes Dev 35:937–939. 10.1101/gad.348691.121 34210804 PMC8247605

[B25] Dastidar SG, Nair D (2022) A Ribosomal Perspective on Neuronal Local Protein Synthesis. Front Mol Neurosci 15:823135. 10.3389/fnmol.2022.823135 35283723 PMC8904363

[B26] Dimitrova-Paternoga L, Jagtap PKA, Cyrklaff A, Vaishali, Lapouge K, Sehr P, Perez K, Heber S, Löw C, Hennig J, Ephrussi A (2021) Molecular basis of mRNA transport by a kinesin-1-atypical tropomyosin complex. Genes Dev 35:976–991. 10.1101/gad.348443.121 34140355 PMC8247599

[B27] Emptage NJ, Carew TJ (1993) Long-term synaptic facilitation in the absence of short-term facilitation in Aplysia neurons. Science 262:253–256. 10.1126/science.8211146 8211146

[B28] Fa M, Staniszewski A, Saeed F, Francis YI, Arancio O (2014) Dynamin 1 is required for memory formation. PLoS One 9:e91954. 10.1371/journal.pone.0091954 24643165 PMC3958425

[B29] Frost WN, Castellucci VF, Hawkins RD, Kandel ER (1985) Monosynaptic connections made by the sensory neurons of the gill-and siphon-withdrawal reflex in Aplysia participate in the storage of long-term memory for sensitization. Proc Natl Acad Sci U S A 82:8266–8269. 10.1073/pnas.82.23.8266 16593630 PMC391484

[B30] Fujimura A, Kishimoto H, Yanagisawa J, Kimura K (2012) Enhancer of rudimentary homolog (ERH) plays an essential role in the progression of mitosis by promoting mitotic chromosome alignment. Biochem Biophys Res Commun 423:588–592. 10.1016/j.bbrc.2012.06.018 22704934

[B31] Fukuda Y, Pazyra-Murphy MF, Silagi ES, Tasdemir-Yilmaz OE, Li Y, Rose L, Yeoh ZC, Vangos NE, Geffken EA, Seo HS, Adelmant G, Bird GH, Walensky LD, Marto JA, Dhe-Paganon S, Segal RA (2021) Binding and transport of SFPQ-RNA granules by KIF5A/KLC1 motors promotes axon survival. J Cell Biol 220:e202005051. 33284322 10.1083/jcb.202005051 PMC7721913

[B32] Fusco CM, Desch K, Dörrbaum AR, Wang M, Staab A, Chan IC, Vail E, Villeri V, Langer JD, Schuman EM (2021) Neuronal ribosomes exhibit dynamic and context-dependent exchange of ribosomal proteins. Nat Commun 12:6127. 10.1038/s41467-021-26365-x 34675203 PMC8531293

[B33] Gallo JM, Spickett C (2010) The role of CELF proteins in neurological disorders. RNA Biol 7:474–479. 10.4161/rna.7.4.12345 20622515 PMC3062235

[B34] Ghirardi M, Montarolo PG, Kandel ER (1995) A novel intermediate stage in the transition between short-and long-term facilitation in the sensory to motor neuron synapse of Aplysia. Neuron 14:413–420. 10.1016/0896-6273(95)90297-x 7857649

[B35] Glater EE, Megeath LJ, Stowers RS, Schwarz TL (2006) Axonal transport of mitochondria requires milton to recruit kinesin heavy chain and is light chain independent. J Cell Biol 173:545–557. 10.1083/jcb.200601067 16717129 PMC2063864

[B36] Gunawardena S, Yang G, Goldstein LS (2013) Presenilin controls kinesin-1 and dynein function during APP-vesicle transport in vivo. Hum Mol Genet 22:3828–3843. 10.1093/hmg/ddt237 23710041 PMC3766177

[B37] Hawkins RD, Lalevic N, Clark GA, Kandel ER (1989) Classical conditioning of the Aplysia siphon-withdrawal reflex exhibits response specificity. Proc Natl Acad Sci U S A 86:7620–7624. 10.1073/pnas.86.19.7620 2798428 PMC298118

[B39] Hirokawa N, Niwa S, Tanaka Y (2010) Molecular motors in neurons: transport mechanisms and roles in brain function, development, and disease. Neuron 68:610–638. 10.1016/j.neuron.2010.09.039 21092854

[B40] Hook V, Yoon M, Mosier C, Ito G, Podvin S, Head BP, Rissman R, O’Donoghue AJ, Hook G (2020) Cathepsin B in neurodegeneration of Alzheimer's disease, traumatic brain injury, and related brain disorders. Biochim Biophys Acta Proteins Proteom 1868:140428. 10.1016/j.bbapap.2020.140428 32305689 PMC7261628

[B41] Jarvela TS, Womack T, Georgiou P, Gould TD, Eriksen JL, Lindberg I (2018) 7B2 chaperone knockout in APP model mice results in reduced plaque burden. Sci Rep 8:9813. 10.1038/s41598-018-28031-7 29955078 PMC6023903

[B42] Kanai Y, Dohmae N, Hirokawa N (2004) Kinesin transports RNA: isolation and characterization of an RNA-transporting granule. Neuron 43:513–525. 10.1016/j.neuron.2004.07.022 15312650

[B43] Kandel ER (2001) The molecular biology of memory storage: a dialogue between genes and synapses. Science 294:1030–1038. 10.1126/science.1067020 11691980

[B44] Kimura T, Watanabe H, Iwamatsu A, Kaibuchi K (2005) Tubulin and CRMP‐2 complex is transported via kinesin‐1. J Neurochem 93:1371–1382. 10.1111/j.1471-4159.2005.03063.x 15935053

[B45] Kosacka J, Nowicki M, Kacza J, Borlak J, Engele J, Spanel‐Borowski K (2006) Adipocyte‐derived angiopoietin‐1 supports neurite outgrowth and synaptogenesis of sensory neurons. J Neurosci Res 83:1160–1169. 10.1002/jnr.20811 16493688

[B46] Ladd AN (2013) CUG-BP, Elav-like family (CELF)-mediated alternative splicing regulation in the brain during health and disease. Mol Cell Neurosci 56:456–464. 10.1016/j.mcn.2012.12.003 23247071 PMC3650117

[B47] Lazarov O, Morfini GA, Pigino G, Gadadhar A, Chen X, Robinson J, Ho H, Brady ST, Sisodia SS (2007) Impairments in fast axonal transport and motor neuron deficits in transgenic mice expressing familial Alzheimer’s disease-linked mutant presenilin 1. J Neurosci 27:7011–7020. 10.1523/JNEUROSCI.4272-06.2007 17596450 PMC2801050

[B48] Lisman J, Yasuda R, Raghavachari S (2012) Mechanisms of CaMKII action in long-term potentiation. Nat Rev Neurosci 13:169–182. 10.1038/nrn3192 22334212 PMC4050655

[B49] Liu XA, Kadakkuzha B, Pascal B, Steckler C, Akhmedov K, Yan L, Chalmers M, Puthanveettil SV (2014) New approach to capture and characterize synaptic proteome. Proc Natl Acad Sci U S A 111:16154–16159. 10.1073/pnas.1401483111 25352669 PMC4234550

[B50] Livak KJ, Schmittgen TD (2001) Analysis of relative gene expression data using real-time quantitative PCR and the 2^−ΔΔCT^ method. Methods 25:402–408. 10.1006/meth.2001.1262 11846609

[B51] Lord C, Ferro-Novick S, Miller EA (2013) The highly conserved COPII coat complex sorts cargo from the endoplasmic reticulum and targets it to the golgi. Cold Spring Harbor Perspect Biol 5:a013367. 10.1101/cshperspect.a013367 PMC3552504 23378591

[B52] Martin KC, Hu Y, Armitage BA, Siegelbaum SA, Kandel ER, Kaang BK (1995) Evidence for synaptotagmin as an inhibitory clamp on synaptic vesicle release in Aplysia neurons. Proc Natl Acad Sci U S A 92:11307–11311. 10.1073/pnas.92.24.11307 7479985 PMC40621

[B53] Martin SJ, Grimwood PD, Morris RG (2000) Synaptic plasticity and memory: an evaluation of the hypothesis. Annu Rev Neurosci 23:649–711. 10.1146/annurev.neuro.23.1.649 10845078

[B54] Mauelshagen J, Parker GR, Carew TJ (1996) Dynamics of induction and expression of long-term synaptic facilitation in *Aplysia*. J Neurosci 16:7099–7108. 10.1523/JNEUROSCI.16-22-07099.1996 8929419 PMC6578949

[B55] Mayford M, Bach ME, Huang YY, Wang L, Hawkins RD, Kandel ER (1996) Control of memory formation through regulated expression of a CaMKII transgene. Science 274:1678–1683. 10.1126/science.274.5293.1678 8939850

[B56] McVicker DP, Awe AM, Richters KE, Wilson RL, Cowdrey DA, Hu X, Chapman ER, Dent EW (2016) Transport of a kinesin-cargo pair along microtubules into dendritic spines undergoing synaptic plasticity. Nat Commun 7:12741. 10.1038/ncomms12741 27658622 PMC5411814

[B57] Monje FJ, Birner‐Gruenberger R, Darnhofer B, Divisch I, Pollak DD, Lubec G (2012) Proteomics reveals selective regulation of proteins in response to memory‐related serotonin stimulation in Aplysia californica ganglia. Proteomics 12:490–499. 10.1002/pmic.201100418 22162403

[B58] Murakoshi H, Shin ME, Parra-Bueno P, Szatmari EM, Shibata AC, Yasuda R (2017) Kinetics of endogenous CaMKII required for synaptic plasticity revealed by optogenetic kinase inhibitor. Neuron 94:37–47.e5. 10.1016/j.neuron.2017.02.036 28318784 PMC5425291

[B59] Nakata T, Hirokawa N (1995) Point mutation of adenosine triphosphate-binding motif generated rigor kinesin that selectively blocks anterograde lysosome membrane transport. J Cell Biol 131:1039–1053. 10.1083/jcb.131.4.1039 7490281 PMC2200001

[B60] Nangaku M, Sato-Yoshitake R, Okada Y, Noda Y, Takemura R, Yamazaki H, Hirokawa N (1994) KIF1B, a novel microtubule plus end-directed monomeric motor protein for transport of mitochondria. Cell 79:1209–1220. 10.1016/0092-8674(94)90012-4 7528108

[B61] Niwa S, Tanaka Y, Hirokawa N (2008) KIF1Bβ-and KIF1A-mediated axonal transport of presynaptic regulator Rab3 occurs in a GTP-dependent manner through DENN/MADD. Nat Cell Biol 10:1269–1279. 10.1038/ncb1785 18849981

[B62] Okada Y, Yamazaki H, Sekine-Aizawa Y, Hirokawa N (1995) The neuron-specific kinesin superfamily protein KIF1A is a unique monomeric motor for anterograde axonal transport of synaptic vesicle precursors. Cell 81:769–780. 10.1016/0092-8674(95)90538-3 7539720

[B63] Otero MG, Alloatti M, Cromberg LE, Almenar-Queralt A, Encalada SE, Pozo Devoto VM, Bruno L, Goldstin LSB, Falzone TL (2014) Fast axonal transport of the proteasome complex depends on membrane interaction and molecular motor function. J Cell Sci 127:1537–1549. 24522182 10.1242/jcs.140780

[B64] Pinsker H, Kupfermann I, Castellucci V, Kandel E (1970) Habituation and dishabituation of the gill-withdrawal reflex in Aplysia. Science 167:1740–1742. 10.1126/science.167.3926.1740 5416541

[B65] Pinsker HM, Hening WA, Carew TJ, Kandel ER (1973) Long-term sensitization of a defensive withdrawal reflex in Aplysia. Science 182:1039–1042. 10.1126/science.182.4116.1039 4748675

[B66] Puthanveettil SV (2013) RNA transport and long-term memory storage. RNA Biol 10:1765–1770. 10.4161/rna.27391 24356491 PMC3917979

[B67] Puthanveettil SV, Monje FJ, Miniaci MC, Choi YB, Karl KA, Khandros E, Gawinowicz MA, Sheetz MP, Kandel ER (2008) A new component in synaptic plasticity: upregulation of kinesin in the neurons of the gill-withdrawal reflex. Cell 135:960–973. 10.1016/j.cell.2008.11.003 19041756 PMC2635114

[B68] Puthanveettil SV, Antonov I, Kalachikov S, Rajasethupathy P, Choi YB, Kohn AB, Citarella M, Yu F, Karl KA, Kinet M, Morozova I, Russo JJ, Ju J, Moroz LL, Kandel ER (2013) A strategy to capture and characterize the synaptic transcriptome. Proc Natl Acad Sci U S A 110:7464–7469. 10.1073/pnas.1304422110 23589870 PMC3645558

[B69] Raimondi A, Ferguson SM, Lou X, Armbruster M, Paradise S, Giovedi S, Messa M, Kono N, Takasaki J, Cappello V, O’Toole E, Ryan TA, De Camilli P (2011) Overlapping role of dynamin isoforms in synaptic vesicle endocytosis. Neuron 70:1100–1114. 10.1016/j.neuron.2011.04.031 21689597 PMC3190241

[B70] Setou M, Seog DH, Tanaka Y, Kanai Y, Takei Y, Kawagishi M, Hirokawa N (2002) Glutamate-receptor-interacting protein GRIP1 directly steers kinesin to dendrites. Nature 417:83–87. 10.1038/nature743 11986669

[B71] Su Q, Cai Q, Gerwin C, Smith CL, Sheng ZH (2004) Syntabulin is a microtubule-associated protein implicated in syntaxin transport in neurons. Nat Cell Biol 6:941–953. 10.1038/ncb1169 15459722

[B72] Sutton MA, Carew TJ (2000) Parallel molecular pathways mediate expression of distinct forms of intermediate-term facilitation at tail sensory-motor synapses in Aplysia. Neuron 26:219–231. 10.1016/s0896-6273(00)81152-6 10798406

[B73] Sutton MA, Schuman EM (2006) Dendritic protein synthesis, synaptic plasticity, and memory. Cell 127:49–58. 10.1016/j.cell.2006.09.014 17018276

[B74] Sutton MA, Masters SE, Bagnall MW, Carew TJ (2001) Molecular mechanisms underlying a unique intermediate phase of memory in Aplysia. Neuron 31:143–154. 10.1016/s0896-6273(01)00342-7 11498057

[B75] Sutton MA, Ide J, Masters SE, Carew TJ (2002) Interaction between amount and pattern of training in the induction of intermediate-and long-term memory for sensitization in Aplysia. Learn Mem 9:29–40. 10.1101/lm.44802 11917004 PMC155928

[B76] Sutton MA, Bagnall MW, Sharma SK, Shobe J, Carew TJ (2004) Intermediate-term memory for site-specific sensitization in *Aplysia* is maintained by persistent activation of protein kinase C. J Neurosci 24:3600–3609. 10.1523/JNEUROSCI.1134-03.2004 15071108 PMC6729755

[B77] Swanwick CC, Shapiro ME, Vicini S, Wenthold RJ (2010) Flotillin‐1 promotes formation of glutamatergic synapses in hippocampal neurons. Dev Neurobiol 70:875–883. 10.1002/dneu.20828 20669324 PMC4482238

[B78] Swarnkar S, Avchalumov Y, Espadas I, Grinman E, Liu XA, Raveendra BL, Zucca A, Mediouni S, Sadhu A, Valente S, Page D, Miller K, Puthanveettil SV (2021) Molecular motor protein KIF5C mediates structural plasticity and long-term memory by constraining local translation. Cell Rep 36:109369. 10.1016/j.celrep.2021.109369 34260917 PMC8319835

[B79] Takeda S, Yamazaki H, Seog DH, Kanai Y, Terada S, Hirokawa N (2000) Kinesin superfamily protein 3 (KIF3) motor transports fodrin-associating vesicles important for neurite building. J Cell Biol 148:1255–1265. 10.1083/jcb.148.6.1255 10725338 PMC2174314

[B80] Tanaka Y, Kanai Y, Okada Y, Nonaka S, Takeda S, Harada A, Hirokawa N (1998) Targeted disruption of mouse conventional kinesin heavy chain kif5B, results in abnormal perinuclear clustering of mitochondria. Cell 93:1147–1158. 10.1016/s0092-8674(00)81459-2 9657148

[B81] Ting L, Rad R, Gygi SP, Haas W (2011) MS3 eliminates ratio distortion in isobaric multiplexed quantitative proteomics. Nat Methods 8:937–940. 10.1038/nmeth.1714 21963607 PMC3205343

[B82] Tong M, Jiang Y (2015) FK506-binding proteins and their diverse functions. Curr Mol Pharmacol 9:48–65. 10.2174/1874467208666150519113541 25986568 PMC6611466

[B83] Tuli L, Ressom HW (2009) LC–MS based detection of differential protein expression. J Proteomics Bioinform 2:416–438. 10.4172/jpb.1000102 20473349 PMC2867618

[B84] Turturici G, Sconzo G, Geraci F (2011) Hsp70 and its molecular role in nervous system diseases. Biochem Res Int 2011:618127. 10.1155/2011/618127 21403864 PMC3049350

[B85] Twelvetrees AE, Yuen EY, Arancibia-Carcamo IL, MacAskill AF, Rostaing P, Lumb MJ, Humbert S, Triller A, Saudou F, Yan Z, Kittler JT (2010) Delivery of GABA_A_Rs to synapses is mediated by HAP1-KIF5 and disrupted by mutant huntingtin. Neuron 65:53–65. 10.1016/j.neuron.2009.12.007 20152113 PMC2841506

[B86] Vakilian H, Mirzaei M, Tabar MS, Pooyan P, Rezaee HL, Parker L, Haynes PA, Gourabi H, Baharvand H, Salekdeh GH (2015) DDX3Y, a male-specific region of Y chromosome gene, may modulate neuronal differentiation. J Proteome Res 14:3474–3483. 10.1021/acs.jproteome.5b00512 26144214

[B87] Wang X, Schwarz TL (2009) The mechanism of Ca2+-dependent regulation of kinesin-mediated mitochondrial motility. Cell 136:163–174. 10.1016/j.cell.2008.11.046 19135897 PMC2768392

[B88] Wobst H, Schmitz B, Schachner M, Diestel S, Leshchyns' ka I, Sytnyk V (2015) Kinesin-1 promotes post-Golgi trafficking of NCAM140 and NCAM180 to the cell surface. J Cell Sci 128:2816–2829. 26101351 10.1242/jcs.169391

[B89] Wozniak MJ, Melzer M, Dorner C, Haring HU, Lammers R (2005) The novel protein KBP regulates mitochondria localization by interaction with a kinesin-like protein. BMC Cell Biol 6:35. 10.1186/1471-2121-6-35 16225668 PMC1266353

[B90] Wu H, Zhou J, Zhu T, Cohen I, Dictenberg J (2020) A kinesin adapter directly mediates dendritic mRNA localization during neural development in mice. J Biol Chem 295:6605–6628. 10.1074/jbc.RA118.005616 32111743 PMC7212647

[B91] Ye X, Carew TJ (2010) Small G protein signaling in neuronal plasticity and memory formation: the specific role of ras family proteins. Neuron 68:340–361. 10.1016/j.neuron.2010.09.013 21040840 PMC3008420

[B92] Zhang C, Liu Y (2017) Retrieving quantitative information of histone PTMs by mass spectrometry. In: Methods in enzymology (Shukla AK, ed), pp 165–191. Cambridge MA: Academic. 10.1016/bs.mie.2016.10.017 28137562

[B93] Zhao C, Takita J, Tanaka Y, Setou M, Nakagawa T, Takeda S, Yang HW, Terada S, Nakata T, Takei Y, Saito M, Tsuji S, Hayashi Y, Hirokawa N (2001) Charcot-Marie-Tooth disease type 2A caused by mutation in a microtubule motor KIF1Bβ. Cell 105:587–597. 10.1016/S0092-8674(01)00363-4 11389829

