# Supplementary figures and images for: Short-Term and Long-Term Sensitization Differentially Alters the Composition of an Anterograde Transport Complex in Aplysia
Source: eNeuro. 2023 Jan 3;10(1):ENEURO.0266-22.2022. doi: 10.1523/ENEURO.0266-22.2022 (PMC9829102; doi:10.1523/ENEURO.0266-22.2022)

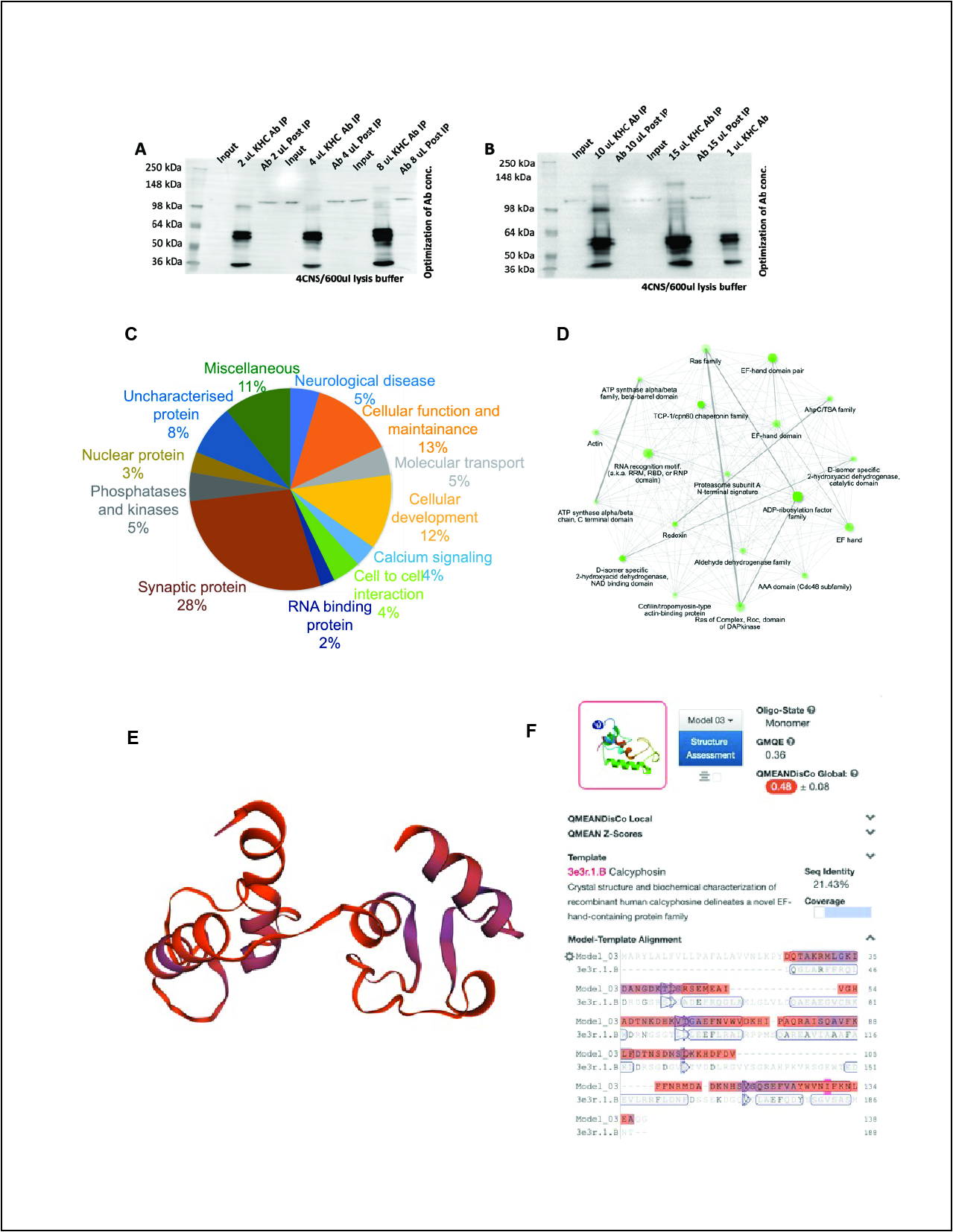

Supplement: Figure 1-2 — A, B, Optimization of ApKHC1 IP with different anti-ApKHC1 antibody concentrations. C, Analysis of proteins that are selectively associated with ApKHC1 kinesin complexes. Protein functions were assigned to a group manually checking their biological role. D, GO network plot of identified kinesin cargo based on protein domains (Pfam). E, Predicted structure of helix-loop-helix EF hand motif uncharacterized protein LOC101861914. F, The structure is based on Template 3e3r.1.B Calcyphosin and showed 21.43% sequence identity with LOC101861914 (www.swissmodel.expasy.org). Download Figure 1-2, TIF file. [file enu-eN-NWR-0266-22-s02.tif]

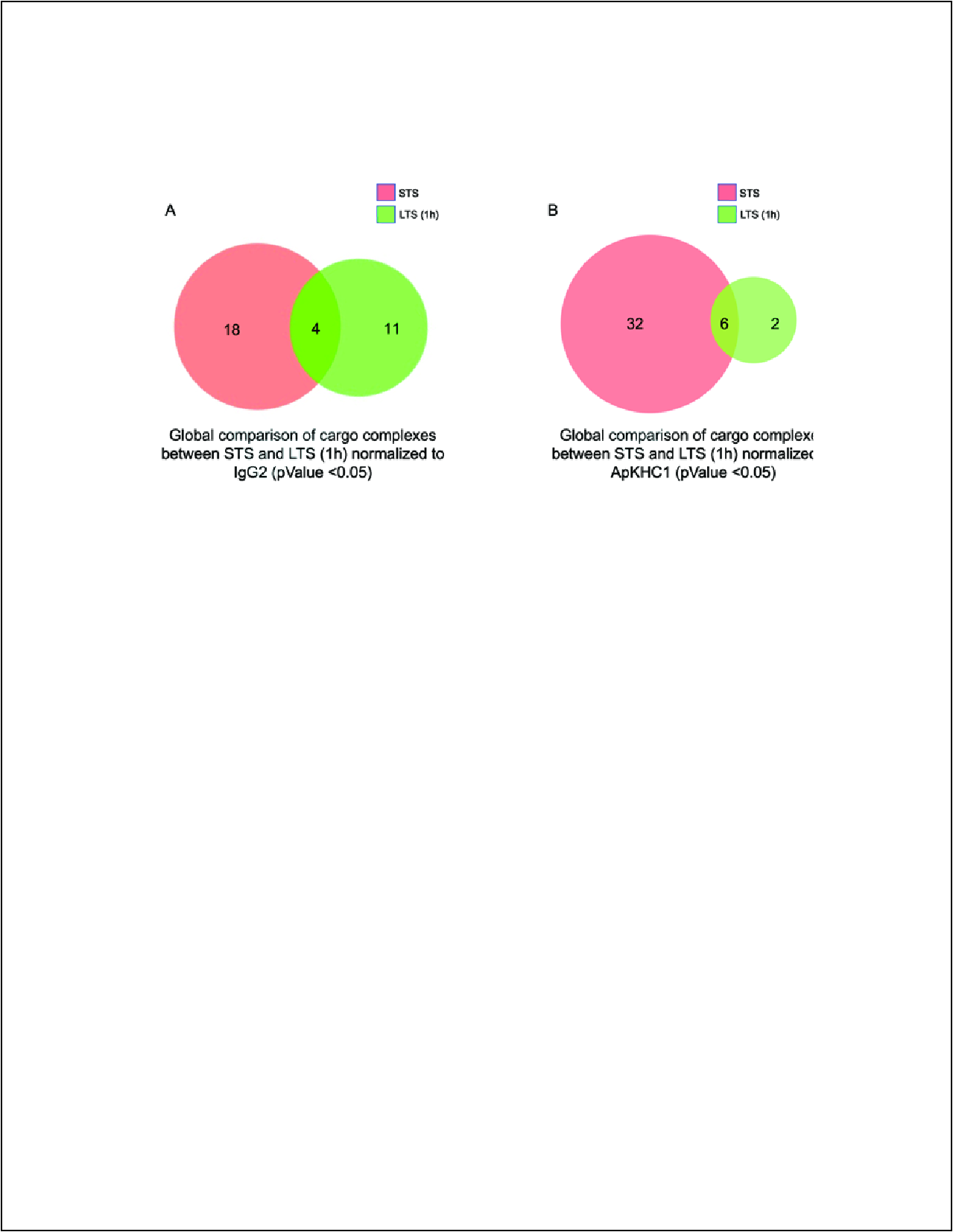

Supplement: Figure 2-2 — Venn diagrams comparing the total number of proteins differentially enriched in ApKHC1 cargos in samples of STS and LTS (1 h). A, Dataset normalized to IgG2. B, Dataset normalized to ApKHC1. Download Figure 2-2, TIF file. [file enu-eN-NWR-0266-22-s03.tif]

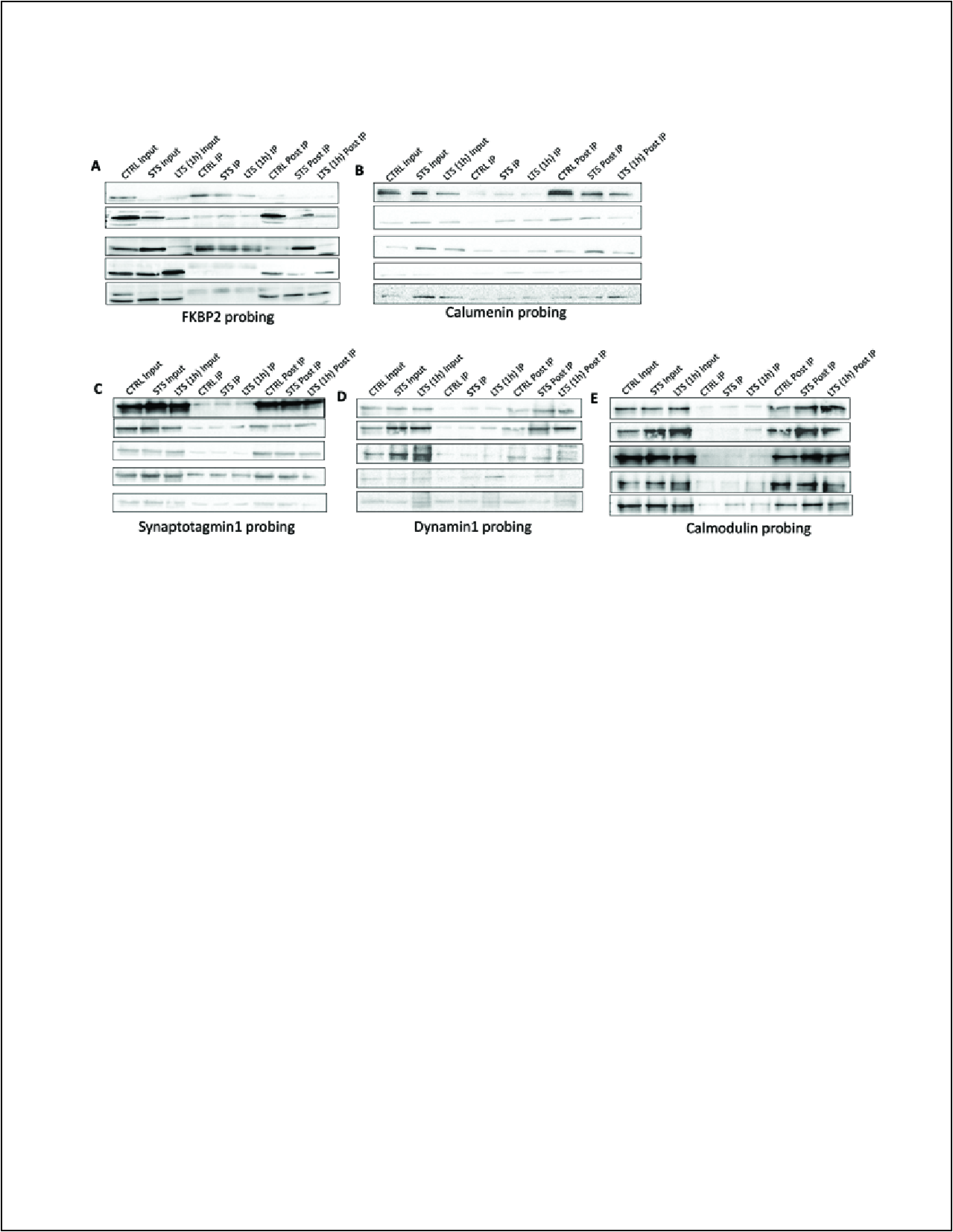

Supplement: Figure 3-2 — Western blots used to measure the band intensities to calculate the relative protein levels of candidates selected from STS and LTS (1 h) training. A, B, FKBP2 (A) and Calumenin (B) are enriched upon STS training. C–E, Synaptotagmin-1 (C), dynamin-1 (D), and calmodulin (E) are enriched in LTS (1 h) training. Download Figure 3-2, TIF file. [file enu-eN-NWR-0266-22-s04.tif]

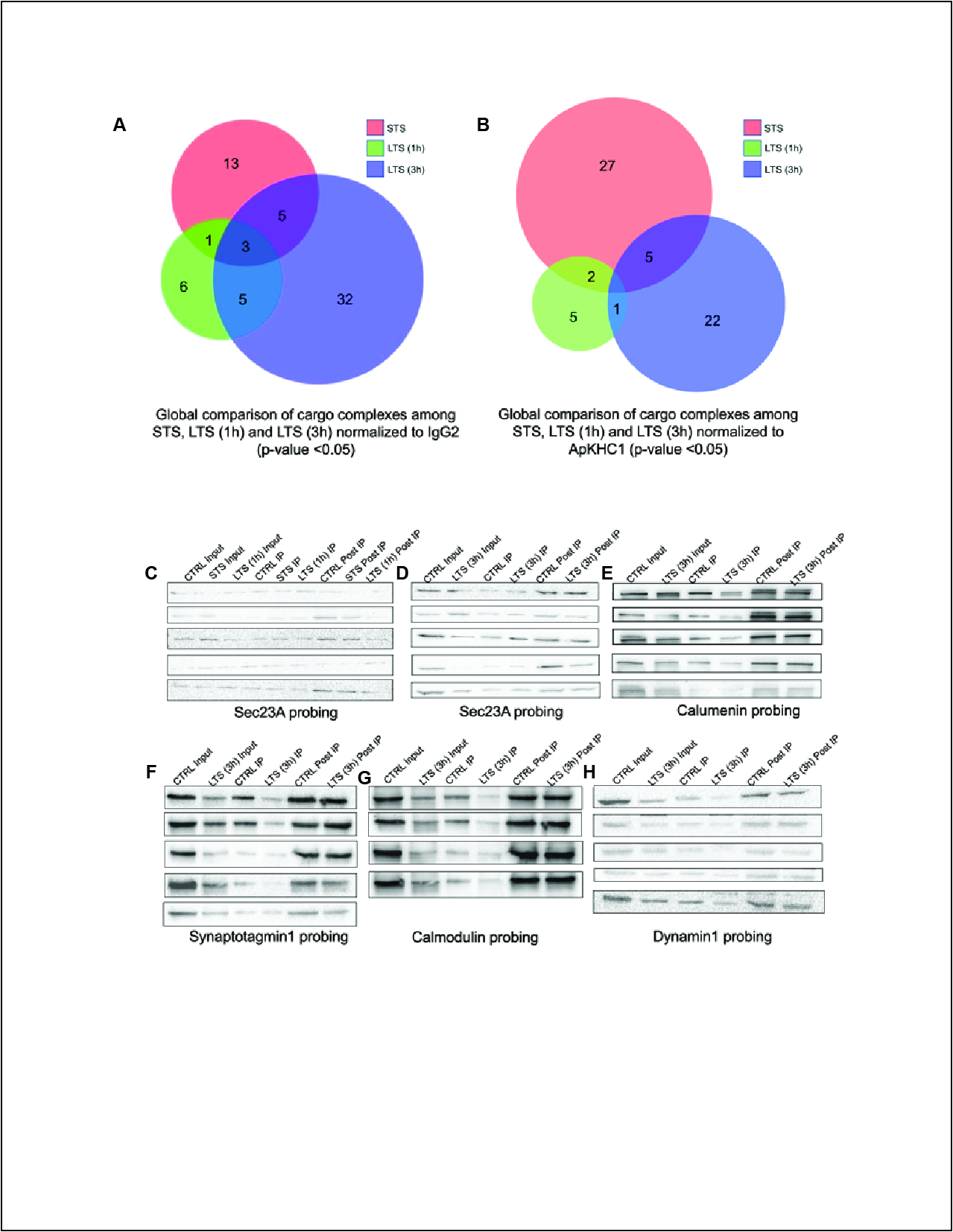

Supplement: Figure 4-2 — A, B, Venn diagrams comparing the total number of proteins differentially enriched in ApKHC1 cargos in samples of STS, LTS (1 h), and LTS (3 h). A, Dataset normalized to IgG2. B, Dataset normalized to ApKHC1. C–H, Western blots used to measure the band intensities to calculate the relative protein levels from candidates selected after 3 h of LTS induction. C, Sec23A enriched after 3 h of LTS and showed no significant difference in enrichment after STS and LTS (1 h). D, However, significant enrichment of sec23A is observed after 3 h of LTS. E–H, Calumenin (E), synaptotagmin-1 (F), calmodulin (G), and dynamin-1 (H) levels were observed to be significantly depleted after 3 h of LTS. Download Figure 4-2, TIF file. [file enu-eN-NWR-0266-22-s05.tif]
